# Targeting the tumor microenvironment in cholangiocarcinoma to improve immune checkpoint blockade: potential strategies and translational pre‐clinical models

**DOI:** 10.1002/cti2.70057

**Published:** 2025-10-24

**Authors:** Maria‐Danae Jessel, Owen McGreevy, Lauryn McReynolds, Lekh N Dahal, Timothy Gilbert, Hassan Z Malik, Christopher E Goldring, Laura E Randle

**Affiliations:** ^1^ The Department of Pharmacology and Therapeutics Institute of Systems, Molecular and Integrative Biology The University of Liverpool Liverpool UK; ^2^ Hepatobiliary Surgery, Liverpool University Hospitals NHS Foundation Trust Royal Liverpool University Hospital Liverpool UK

**Keywords:** cholangiocarcinoma, human, immunotherapy, preclinical, tumor microenvironment

## Abstract

Cholangiocarcinoma is a malignancy of significant unmet clinical need with limited therapeutic options. Most patients are diagnosed at advanced or metastatic stages, where surgical resection with curative intent is no longer an option. Gemcitabine‐cisplatin chemotherapy has been the standard of care for these patients, remaining unchanged for over a decade. Recently, the addition of the programmed death ligand 1 inhibitor, durvalumab, to this regimen demonstrated an objective response rate of 26.7% in a phase III trial, becoming the new standard of care for advanced cholangiocarcinoma. Although considered a success in cholangiocarcinoma treatment, the results indicate that only a small proportion of patients respond to treatment with immune checkpoint inhibitors. Emerging evidence suggests that many cholangiocarcinoma tumors exhibit an immunologically ‘cold’ tumor microenvironment, characterised by predominance of immunosuppressive immune populations and limited infiltration of cytotoxic T cells, which contributes to their resistance to immune checkpoint inhibitors. This review provides a comprehensive overview of the research studies that have employed immunomodulatory strategies in cholangiocarcinoma aimed at priming the tumor microenvironment for a more effective response to immune checkpoint inhibitors. This update will also evaluate the strengths and limitations of current pre‐clinical models of cholangiocarcinoma, with emphasis on more advanced translational models. These complex models remain underutilised, hindering the development of novel therapeutic approaches. We suggest that these complex preclinical models may help translation of therapies into clinical practice.

## Graphical abstract

Cholangiocarcinoma is a challenging malignancy with limited treatment options, where most patients are diagnosed at advanced stages. While the addition of durvalumab to gemcitabine‐cisplatin has improved outcomes, only a small subset of patients responds because of an immunosuppressive tumor microenvironment. This review explores immunomodulatory strategies to enhance immune checkpoint inhibitor efficacy and evaluates advanced pre‐clinical models that could improve therapeutic translation.

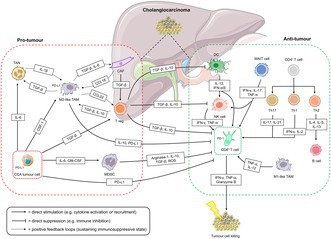

## Introduction

Cholangiocarcinoma (CCA) is an invasive adenocarcinoma of the biliary tract, classified into three groups based on anatomical origin: intrahepatic (iCCA), perihilar (pCCA) and distal (dCCA).^1^, ^2^ iCCA arises in the periphery of the second‐order bile ducts, while pCCA and dCCA, together referred to as extrahepatic CCA (eCCA), originate in the left, right or common hepatic ducts and the common bile duct, respectively.^1^, ^3^ Although only iCCA is anatomically defined as a primary liver cancer, many epidemiological studies group all CCA subtypes together, with CCA collectively regarded as the second most common primary liver cancer after hepatocellular carcinoma (HCC), accounting for ~15% of cases.^1^, ^4^, ^5^ This broader classification, still used in several studies and reflected in historical cancer registry records of biliary tract tumors, is now considered outdated because of emerging distinctions between intrahepatic and extrahepatic subtypes.^6^, ^7^ These developments contributed to the adoption of a three‐subtype CCA classification in the 11th revision of the International Classification of Diseases by the World Health Organization.^8^

**Figure 1:**
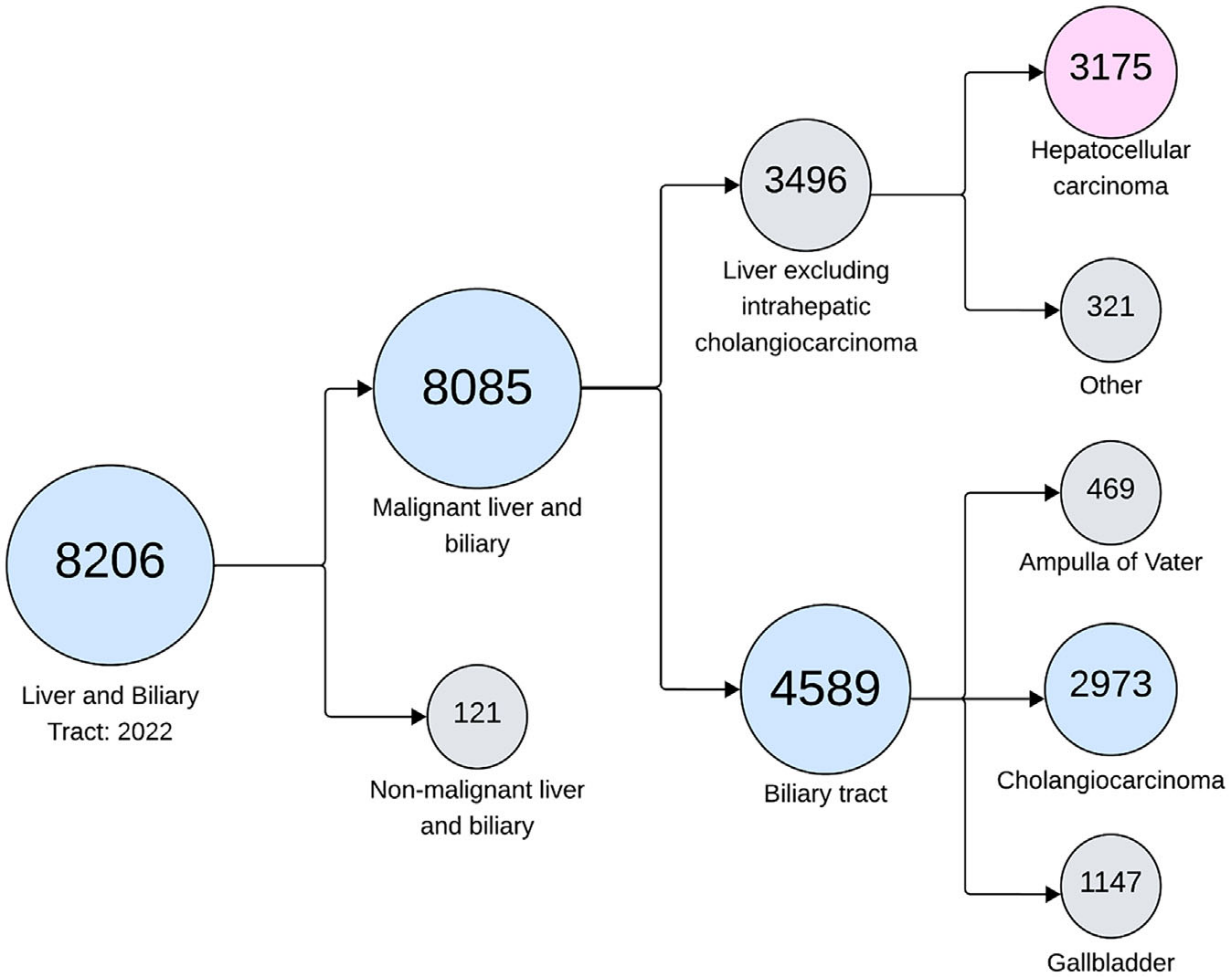
Tree diagram visualising UK data on liver and biliary tract cancer diagnoses in 2022, from the NHS Get Data Out programme (National Disease Registration Service, NDRS). A total of 8206 cases of liver and biliary tract conditions were recorded in the United Kingdom in 2022. These were classified into malignant cases (*n* = 8085) and non‐malignant cases (*n* = 121). Malignant cases were further divided into liver cancers excluding intrahepatic cholangiocarcinoma (iCCA) (*n* = 3496) and biliary tract cancers (*n* = 4589). Liver cancers were subdivided into hepatocellular carcinoma (HCC, *n* = 3175) and other liver cancer types (*n* = 321). Biliary tract cancers were classified into cholangiocarcinoma (CCA, *n* = 2973), gallbladder cancer (*n* = 1147), and ampulla of Vater cancer (*n* = 469). Notably, the number of CCA cases (*n* = 2973) was nearly equivalent to the number of HCC cases (*n* = 3175) in 2022, highlighting a shift in incidence that underscores the growing clinical relevance of CCA in the United Kingdom. Adapted from: NDRS, Get Data Out programme (2022).^9^

The global incidence and mortality of CCA, especially iCCA, have risen in recent years.^1^ While incidence and mortality vary significantly by region, with the highest rates of iCCA reported in Eastern Asia, Western countries are experiencing a concerning increase.^1^ Notably, data from the UK National Disease Registration Service's Get Data Out programme suggest that in 2022, the number of CCA diagnoses nearly equalled HCC diagnoses,^9^ underscoring the urgent need for intensified CCA research to address this growing health burden (Figure 1). Despite growing interest, CCA remains a cancer of significant unmet clinical need, demonstrated by its rising incidence and poor 5‐year overall survival (OS) of just 7–20% in advanced or unresectable cases.^10^

This review provides a comprehensive update on recent advancements in CCA treatment, with a focus on immunotherapy and the tumor microenvironment (TME) as a key determinant of therapeutic response. We discuss key research efforts aimed at improving immunotherapy efficacy, emphasise the need for more translationally relevant preclinical models to study CCA TME dynamics and explore how these models may guide the development of more effective therapeutic combinations. We outline future directions for enhancing immunotherapy outcomes and highlight critical translational gaps that warrant further investigations.

## Advances in cholangiocarcinoma chemo‐immunotherapy

Currently, the only potentially curative treatment for CCA is radical surgical resection; however, up to 80% of patients present with unresectable or metastatic disease.^11^ Even after surgery, recurrence rates remain high, and the 5‐year OS is limited to 20–40% in most series.^12^ As a result, various adjuvant modalities have been explored to improve survival post‐resection.

Since 2017, three randomised phase III trials have assessed the effect of adjuvant chemotherapy on patient outcomes, with mixed results. The BCAT study (UMIN‐CTR; ID 000000820) randomised patients with resected CCA to observation or gemcitabine, finding no significant survival probability between groups.^13^ Similarly, the PRODIGE12‐ACCORD18 trial (EudraCT; 2008‐004560‐39) assessed gemcitabine–oxaliplatin in patients with resected CCA, reporting no significant difference in relapse‐free survival (RFS) and OS between treatment and observation groups.^14^ In contrast, the BILCAP trial (NCT00363584) demonstrated significantly improved OS and RFS with adjuvant capecitabine in the per‐protocol population, showing a clinically meaningful OS benefit of 14.7 months.^15^ Based on these results, the American Society of Clinical Oncology recommends 6 months of adjuvant capecitabine as the standard of care following CCA resection.^16^

For patients with unresectable or metastatic CCA, combination chemotherapy remains the primary treatment, alongside trials investigating targeted molecular therapies and immunotherapies. The ABC‐02 phase III trial (NCT00262769) compared gemcitabine–cisplatin to gemcitabine monotherapy in 410 patients with advanced CCA, gallbladder cancer or ampullary cancer.^17^ Dual chemotherapy increased median OS by 3.6 months and progression‐free survival by 3 months, compared to gemcitabine alone.^17^ Although the median OS remained under 1 year, this represented a major improvement at the time, leading to gemcitabine–cisplatin being established as the first‐line treatment for advanced CCA, as recommended by the National Institute for Health and Care Excellence (NICE) guidelines until early 2024.^18^ The standard of care for unresectable CCA remained unchanged for over 10 years.^19^

CCAs display immunogenic characteristics, including expression of immune checkpoint molecules such as programmed death‐ligand 1 (PD‐L1) and cytotoxic T‐lymphocyte‐associated protein 4 (CTLA‐4) within the TME.^20^, ^21^, ^22^ Chemotherapy has been shown to exert immunomodulatory effects that may enhance antitumor immunity.^23^, ^24^ These observations provided the rationale for combining immune checkpoint inhibitors (ICIs) with standard chemotherapy to improve patient outcomes.

A phase II trial (NCT03046862) evaluating durvalumab (PD‐L1 inhibitor) plus gemcitabine–cisplatin showed a promising objective response rate (ORR) of 72% and median OS of 20.2 months, with no dose‐limiting toxicity, providing a proof of concept for this approach.^25^ This led to the TOPAZ‐1 phase III trial (NCT03875235) evaluating durvalumab plus gemcitabine–cisplatin versus placebo plus gemcitabine–cisplatin in 685 patients with advanced CCA.^19^ Durvalumab significantly improved OS (12.8 months versus 11.5 months) and ORR (26.7% versus 18.7%) compared to chemotherapy alone.^19^ Consequently, gemcitabine–cisplatin–durvalumab became the new first‐line treatment for advanced CCA,^26^ replacing chemotherapy alone in NICE guidelines.^18^ However, the modest ORR highlights that only a small subset of patients derives meaningful clinical benefit, emphasising important gaps in our understanding of ICI response predictors and resistance mechanisms.

The KEYNOTE‐966 phase III trial (NCT04003636) assessed a programmed death 1 (PD‐1) inhibitor pembrolizumab plus gemcitabine–cisplatin in 1069 patients with unresectable CCA.^27^ The pembrolizumab group had a median OS benefit of 1.8 months over the chemotherapy‐alone group.^27^ The authors concluded that, given the statistically significant OS improvement and acceptable safety profile, pembrolizumab plus gemcitabine–cisplatin represents a viable treatment option for previously untreated metastatic or unresectable CCA.^27^ However, NICE has not yet updated the guidelines to include pembrolizumab for advanced CCA,^28^ leaving uncertainty over its routine use.

A summary of completed and ongoing ICI trials, including primary endpoints, is provided in Table 1. This review includes only trials evaluating ICI–chemotherapy combinations.

**Table 1:** Summary of immune checkpoint inhibitor (ICI)‐based treatment strategies evaluated in clinical trials for biliary tract cancer (BTC) and other selected tumor types

Treatment approach	Treatment/Intervention	Trial identifier	Phase	Target (s)	Cohort	ORR (%)	OS (months)	Ocurrence of Grade 3/4 AEs (%)	Current Status
ICI monotherapy	Atezolizumab ± Cobimetinib	NCT03201458 ^29^	II	PD‐L1	BTC	2.8	NA	38.5	Completed
	Durvalumab ± Tremelimumab	NCT01938612 ^30^	I	PD‐L1	BTC	4.8	8.1	19	Completed
	Nivolumab	NCT02829918 ^31^	II	PD‐1	BTC	22	14.2	17	Completed
	Pembrolizumab	NCT02054806 KEYNOTE‐028^32^	Ib	PD‐1	Solid Tumors	13	5.7	16.7	Completed
	Pembrolizumab	NCT02628067 KEYNOTE‐158^32^	II	PD‐1	MMR deficient non‐colorectal cancers	5.8	7.4	13.5	Recruiting
ICI combination	Durvalumab & Temelimumab ± Capecitabine	NCT05239169^33^	II	PD‐L1+ CTLA‐4	BTC	10.8	10.1	23.1	Completed
	Ipillimab & Nivolumab	NCT02923934 ^34^	II	CTLA‐4 + PD‐1	BTC	23	5.7	15	Completed
	Nivolumab & Ipilimumab/Gem‐Cis	NCT03101566 ^35^	II	CTLA‐4 + PD‐1	BTC	NA	8.2	15	Completed
	Vudalimab	NCT05297903^36^	II	CTLA‐4 + PD‐1	BTC	NA	NA	NA	Active, not recruiting
ICI + Chemotherapy	Camrelizumab & Gem‐Ox	NCT03486678 ^37^	II	PD‐1	BTC	54	11.8	70	Completed
	Gem‐Cis ± Durvalumab	NCT03875235 TOPAZ‐1^19^	III	PD‐L1	BTC	26.7	12.8	75.7	Active, not recruiting
	Nivolumab ± Gem‐Cis	JapicCTI‐153 098^38^	I	PD‐1	BTC	36.7	15.4	77	Completed
	Nivolumab & Gem‐Cis	NCT03311789 ^39^	II	PD‐1	BTC	55.6	8.5	NA	Unknown
	Gem‐Cis ± Pembrolizumab	NCT04003636 KEYNOTE‐966^27^	III	PD‐1	BTC	29	12.7	77	Completed
	Gem‐Cis ± Envafolimab	NCT04910386^40^	II	PD‐L1	BTC	NA	NA	NA	Not yet recruiting

This table presents an overview of clinical trials assessing ICI monotherapy, ICI combinations, and ICI combined with chemotherapy in patients with BTC and related solid tumors. For each treatment regimen, the trial identifier, phase, targeted immune checkpoint(s), patient cohort, objective response rate (ORR), overall survival (OS) and incidence of grade 3/4 adverse events (AEs) are provided. Trials include various PD‐1/PD‐L1 and CTLA‐4 targeting agents, either alone or in combination with each other and/or standard chemotherapeutics (e.g. gemcitabine and cisplatin). OS and AE data are reported in months and percentages, respectively. ‘NA’ indicates data not available. Trials are categorised by treatment approach. The current recruitment or completion status is noted for each study. Cohorts beyond BTC include solid tumors and mismatch repair (MMR)‐deficient non‐colorectal cancers.

ICI therapy has revolutionised oncology, demonstrating exceptional efficacy in some patients and driving advances in patient stratification and understanding of tumor immunology.^41^ However, many tumors, including CCA, exhibit resistance to ICIs. Proposed mechanisms include tumor‐intrinsic factors, multiple immune checkpoint co‐expression, aetiology‐dependent mechanisms, host factors and various metabolites.^42^ The TME plays a central role in modulating antitumor immune responses throughout the course of treatment and is a key determinant of patient response to ICIs.^43^

## Tumor microenvironment of cholangiocarcinoma

**Figure 2:**
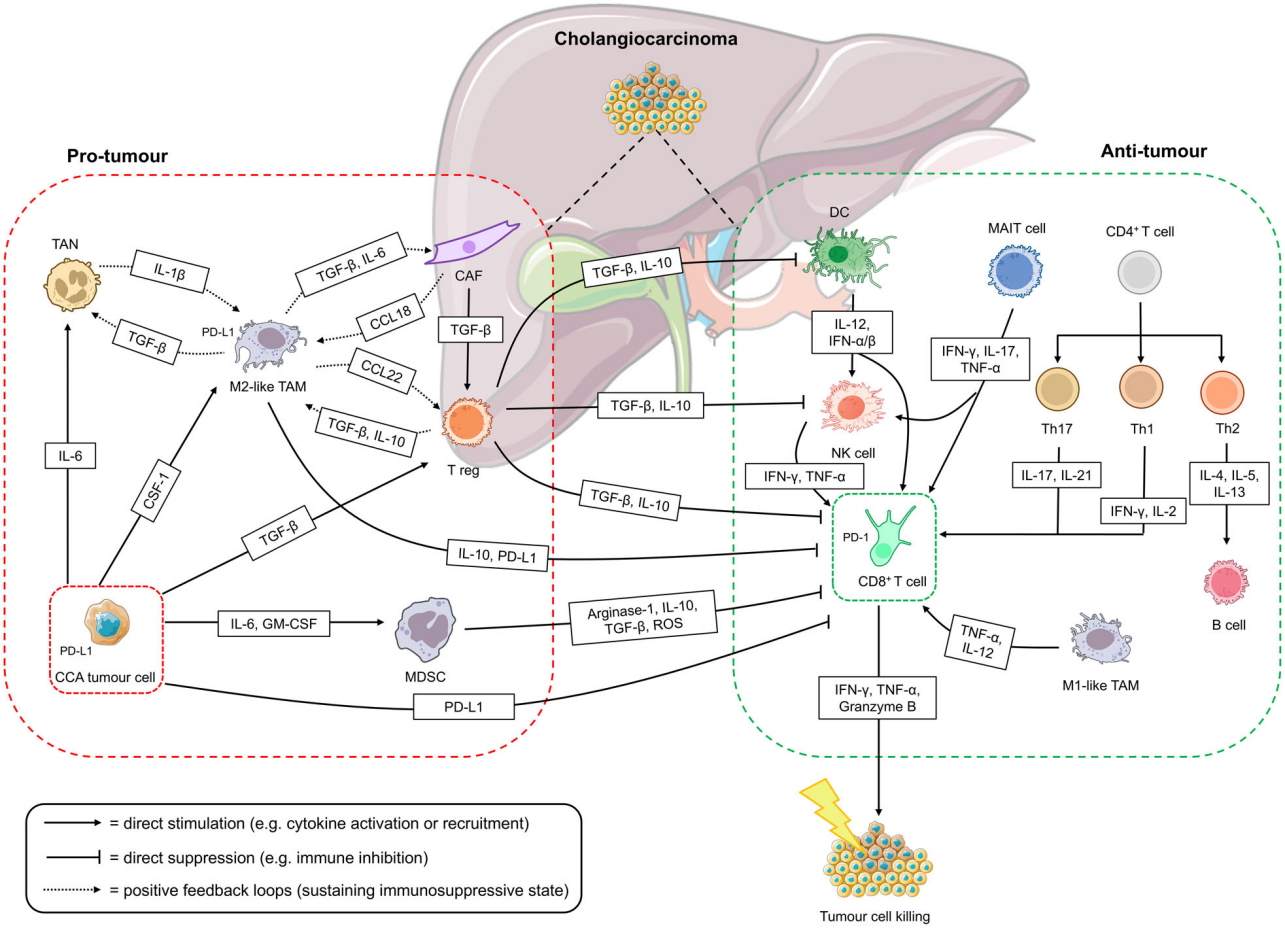
Immunological landscape of the CCA TME, highlighting pro‐tumor and antitumor immune interactions. The left panel (red dotted box) illustrates the tumor‐promoting compartment, consisting of CCA tumor cells, cancer‐associated fibroblasts (CAFs), M2‐like tumor‐associated macrophages (TAMs), regulatory T cells (Tregs), tumor‐associated neutrophils (TANs) and myeloid‐derived suppressor cells (MDSCs). These cells secrete immunosuppressive cytokines such as TGF‐β, IL‐10, IL‐6 and CCL22, and express PD‐L1, contributing to T‐cell dysfunction and immune evasion. Tumor‐derived factors including TGF‐β, CSF1, IL‐6 and GM‐CSF activate and recruit these suppressive cells. Several positive feedback loops (dotted black lines) between CAFs, TAMs, Tregs and TANs amplify immunosuppression and fibrotic remodelling. The right panel (green dotted box) shows the antitumor immune compartment, including CD8^+^ cytotoxic T cells, natural killer (NK) cells, dendritic cells (DCs), T helper 1 (Th1) and 17 (Th17), mucosal‐associated invariant T (MAIT) cells, and B cells. These cells promote tumor cell killing through the release of IFN‐γ, TNF‐α, IL‐2, IL‐12, IL‐17 and granzyme B. Dendritic cells initiate T‐cell responses through antigen presentation and cytokines (IL‐12, IFN‐α/β), while Th1 and MAIT cells support CD8^+^ T cell and NK cell activity. However, their function is frequently inhibited by suppressive cytokines and PD‐L1 expressed on tumor and stromal cells. Together, this diagram illustrates the dynamic and highly suppressive TME in CCA, where tumor‐promoting cell networks undermine cytotoxic immune responses and promote disease progression.

The CCA TME is characterised by a highly desmoplastic stroma and abundant immunosuppressive immune cells. Cancer‐associated fibroblasts (CAFs) and the extracellular matrix (ECM) they produce interact with various cell types, influencing tumor growth, nutrient supply, immune modulation and providing immune barriers.^44^, ^45^ Persistently activated CAFs secrete tumor‐promoting cytokines such as transforming growth factor‐β (TGF‐β), platelet‐derived growth factor and insulin‐like growth factors I/II, which promote tumor proliferation, immune evasion and resistance to apoptosis.^46^, ^47^ High tumor CAF levels in CCA correlate with poor prognosis.^48^ The CCA TME has a high infiltration of immunosuppressive myeloid cells, specifically, myeloid‐derived suppressor cells (MDSCs), tumor‐associated macrophages (TAMs), tumor‐associated neutrophils (TANs) and regulatory T cells (Tregs)^49^, ^50^, ^51^; and hence, a dampened antitumor immune response^51^ (Figure 2).

### Pro‐tumor immunity in CCA

TAMs, predominantly exhibiting an M2‐like phenotype, are abundant in CCA and promote tumor growth, angiogenesis, epithelial–mesenchymal transition and immunosuppression. CCA tumor cells drive M2 polarisation through cytokines such as IL‐6 and TGF‐β, acting via the IL‐6/STAT3 pathway.^52^ TAMs secrete immunosuppressive factors, including interleukin‐10 (IL‐10), vascular endothelial growth factor (VEGF), granulocyte–macrophage colony‐stimulating factor (GM‐CSF), tumor necrosis factor‐α (TNF‐α) and chemokines such as chemokine (C‐C motif) ligand (CCL)17 and CCL22, which recruit Tregs, MDSCs and TANs to the TME.^53^, ^54^ They also produce Wnt ligands (Wnt3a and Wnt7b), supporting tumor proliferation via canonical Wnt signalling.^55^, ^56^ TAM density correlates with tumor grade, microvascular density and extrahepatic metastasis in iCCA and eCCA.^52^, ^54^, ^57^ Attempts to target TAMs, such as blocking colony‐stimulating factor 1 receptor (CSF1R), have shown limited success because of compensatory MDSC infiltration via chemokine (C‐X‐C motif) ligand (CXCL)2, sustaining immune suppression.^58^

MDSCs further amplify immunosuppression, although their phenotypes and recruitment mechanisms in CCA are less well‐defined. Tumor‐derived GM‐CSF, IL‐6 and VEGF‐A attract MDSCs, which inhibit T‐cell responses via TGF‐β, IL‐10, arginase (ARG)‐1 and reactive oxygen species.^58^, ^59^

TANs exhibit functional plasticity, adopting either pro‐tumor (N2) or antitumor (N1) phenotypes. TGF‐β promotes N2 polarisation, enhancing tumor progression.^60^ TAN recruitment is mediated by CXCL5 from CCA cells, activating PI3K–Akt and ERK1/2–MAPK pathways to support tumor growth and metastasis.^61^ TAN infiltration correlates with reduced OS in both iCCA and eCCA.^49^, ^62^, ^63^ TANs and TAMs engage in reciprocal crosstalk; TAN‐derived IL‐1β stimulates TAMs, while TAMs release IL‐11 and oncostatin M, reinforcing TAN‐mediated tumor growth.^64^ In eCCA, high TAN levels associate with increased Treg infiltration and decreased CD8^+^ T cells, contributing to an immunosuppressive TME.^49^

Tregs, particularly Forkhead box P3 (FOXP3)^+^ subsets, contribute to T‐cell dysfunction. A high FOXP3^+^:CD8^+^ T‐cell ratio predicts reduced OS.^65^ Tumor‐derived IL‐2 and TGF‐β convert CD4^+^CD25^−^ cells into Tregs.^66^ Tregs suppress CD8^+^ T cells and are linked to worse prognosis and lymphatic metastasis, particularly in eCCA, even post‐resection.^49^, ^67^ FOXP3 downregulation reduces tumor proliferation, invasion and immunosuppressive cytokine signalling, indicating a functional role in tumor progression and immune evasion.^68^ Tregs suppress CD8^+^ T cells, natural killer (NK) cells and dendritic cells (DCs), establishing an immune‐permissive niche.

### Antitumor immunity in CCA

NK cells, though abundant in the liver and capable of mediating cytotoxicity through granzyme B, FasL and TNF‐α, are often impaired in CCA patients because of alterations of the killer cell immunoglobulin‐like receptor and human leukocyte antigen gene loci.^69^ Tumor‐induced shedding of the stress ligand MICA/B blocks NKG2D‐mediated tumor recognition, further restricting NK cell function.^70^ However, high CXCL9 expression within tumors promotes NK cell infiltration and correlates with improved outcomes.^71^ Preclinical studies demonstrate that adoptive transfer of expanded or activated NK cells suppresses tumor growth, particularly when combined with monoclonal antibodies such as cetuximab.^72^, ^73^ Neutralising antibodies against soluble MICA/B restore NK cell degranulation and IFN‐γ production in co‐culture with CCA cells.^70^

DCs are antigen‐presenting cells that initiate adaptive immunity by processing and presenting tumor‐associated antigens to naïve T cells.^74^ In CCA, mature CD83^+^ DCs are commonly found at the tumor periphery alongside CD4^+^ and CD8^+^ T cells, suggesting a role in bridging innate and adaptive immune responses, although their peripheral localisation may reflect immune exclusion.^75^, ^76^ Increased mature DC infiltration is associated with better prognosis and fewer lymph node metastases.^75^ However, tumor‐derived TGF‐β and IL‐10 impair DC maturation and function, reducing T‐cell activation.^77^ Engineered monocyte‐derived DCs enhance CD8^+^ T‐cell cytotoxicity *in vitro*,^78^ and pro‐inflammatory DCs promote Th1 responses and tumor‐specific cytotoxicity in CCA.^79^ Nonetheless, the dual immunostimulatory and immunosuppressive functions of DCs complicate their clinical application in CCA.

Mucosal‐associated invariant T (MAIT) cells, which typically reside in liver tissue, also affect CCA prognosis. Patients with higher hepatic MAIT cell levels show improved OS, suggesting a protective role.^51^ CD4^+^ T helper (Th) cells coordinate adaptive immune responses in CCA, particularly at the tumor border,^80^ where they support B cell activity.^22^ Th cells differentiate into subsets with distinct cytokine profiles: Th1 cells produce IFN‐γ and IL‐2, promoting cytotoxic immunity; Th2 cells secrete IL‐4, IL‐5 and IL‐13, contributing to humoral responses; and Th17 cells generate IL‐17 and IL‐22, which can be either tumor‐promoting or ‐suppressive depending on context.^81^, ^82^, ^83^, ^84^ In one case, adoptive transfer of mutation‐specific Th1 cells stabilised metastatic CCA lesions, highlighting the therapeutic potential of Th cells.^85^

CD8^+^ T cells are central to adaptive anti‐tumor immunity in CCA, executing cytotoxicity through perforin, granzyme B, FasL and TNF‐α.^86^ In one study, over 50% of resected CCAs showed CD8^+^ tumor‐infiltrating lymphocytes (TILs), with approximately 30% expressing granzyme B, indicating functional activity.^87^ High granzyme B^+^ CD8^+^ T cell density is associated with improved OS in both iCCA and dCCA.^88^ Sabbatino *et al*.^21^ reported significantly greater CD8^+^ T cell infiltration in fibrous septa than tumor lobules. They linked this reduced infiltration to poor responses to ICIs, that is tumors lacking CD8^+^ T cells do not respond well to immunotherapy.^21^ CD8^+^ T cells can also induce PD‐L1 expression on tumor cells, contributing to adaptive resistance.^89^ Nevertheless, elevated CD8^+^ T cell infiltration correlates with lower recurrence, better outcomes^90^, and is enriched in lymphoepithelioma‐like and Epstein–Barr virus‐associated CCA, where it is a favorable prognostic marker.^91^

Understanding how these innate and adaptive immune populations interact within the TME, and how tumor‐derived cytokines modulate them, remains critical to designing rational immunotherapeutic strategies. The profound immune suppression seen in CCA presents both a barrier to ICI efficacy and an opportunity for multi‐target interventions.

## Immune phenotype of cholangiocarcinoma

Hegde *et al*.^92^ proposed a tumor immunity continuum comprising three immune phenotypes based on the spatial distribution of CD8^+^ T cells within the TME, each associated with distinct responses to immunotherapy. These phenotypes are as follows: (1) inflamed tumors; (2) immune‐excluded tumors; and (3) immune desert tumors.^92^, ^93^ Inflamed tumors have robust CD8^+^ T cell infiltration, intact antigen presentation, increased interferon‐γ (IFN‐γ) signalling, high tumor mutational burden (TMB) and elevated PD‐L1 expression.^92^ Also known as ‘hot’ tumors, they are generally more responsive to ICIs.^94^ In contrast, immune‐excluded tumors have expanded but poorly infiltrated CD8^+^ T cells, low TMB, low PD‐L1 expression, increased tumor angiogenesis, and an accumulation of immunosuppressive cells such as TAMs, MDSCs and Tregs.^92^, ^95^ Immune desert tumors lack CD8^+^ T cells and, like immune‐excluded tumors, are resistant to PD‐1/ PD‐L1 blockade.^92^, ^95^ These ‘cold’ tumors are characterised by immunological ignorance, including low major histocompatibility complex I presentation and poor tumor antigen presentation, Treg‐mediated tolerance, and insufficient T cell priming and activation, contributing to ICI resistance.^93^

Although many studies assess CCA as a unified a entity, emerging evidence suggests meaningful immunological differences between iCCA and eCCA subtypes. Using bulk gene expression profiling, Job *et al*.^96^ identified four immune subtypes of iCCA. Nearly half (46%) of the tumors fell into the immune desert group, characterised by minimal immune infiltration and poor activation. Only 13% displayed an inflamed phenotype marked by high CD8^+^ T cell infiltration and active immune checkpoint signalling, associated with the longest median survival of 73 months, compared to 42 months for the immune desert group.^96^ The remaining subtypes included a myeloid‐rich group (19%) with moderate immune cell presence but low lymphoid activity, and a mesenchymal subtype (22%) with high stromal and ECM‐related gene expression.^96^ The latter subtypes had poorer median survival (25 and 19 months, respectively) and may represent immunotherapy‐resistant populations.^96^

In a separate study using multiplex immunohistochemistry and gene expression profiling of 104 CCA samples (all subtypes), iCCA tumors had higher densities of non‐exhausted CD8^+^ T cells, which correlated with improved survival.^88^ Conversely, eCCA subtypes showed lower CD8^+^ T cell infiltration and enrichment of PD‐L1^+^ TAMs, associated with worse outcomes.^88^ Kitano *et al*.^49^ also found that eCCA is frequently infiltrated by TANs and Tregs, which negatively correlate with CD8^+^ T cells and are linked to poor prognosis.

To date, few studies have directly compared the TME of iCCA and eCCA subtypes, with most immunological analyses focussed on iCCA. This represents a critical gap, as subtype‐specific immune profiles may significantly influence immunotherapy responsiveness. Future studies should prioritise comparative TME analyses to guide personalised therapeutic approaches in CCA.

## Mechanisms of immunotherapy resistance

The multiple disease subtypes, desmoplastic stroma and immunosuppressive TME of CCA are believed to contribute to the ICI resistance seen in this cancer type.^97^ Better understanding of the composition and interactions within the TME is essential for identifying the most effective immunotherapeutic strategies for CCA patients. Resistance to immunotherapy involves both tumor‐intrinsic and ‐extrinsic mechanisms.^98^ Tumor‐intrinsic factors include alterations in antitumor immune signalling pathways, such as the IFN‐γ pathway,^98^ which upregulates PD‐L1 expression on tumor cells to aid immune escape.^99^ Tumor cells may also secrete exosomal PD‐L1 to peripheral blood to inhibit T cell function systemically^100^ and exhibit oncogenic mutations (e.g. mitogen‐activated protein kinase and epidermal growth factor receptor pathway alterations),^98^ which contribute to immunosuppression. Extrinsic factors include the presence of immunosuppressive cell populations (e.g. MDSCs, Tregs and TAMs) within the local TME, along with host‐related factors such as age, sex, microbiome, genetics and the environment.^98^

Among the key immunosuppressive populations in the CCA TME are MDSCs and TAMs. MDSCs, myeloid in origin, can be either granulocytic/polymorphonuclear (G‐MDSCs or PMN‐MDSCs) or monocytic (M‐MDSCs), which resemble neutrophils and monocytes, respectively.^101^, ^102^ MDSCs contribute to tumor progression by promoting immune evasion, angiogenesis, proliferation, invasion and metastasis.^103^ These cells inhibit T cell function through the production of cytokines (e.g. TGF‐β, IL‐10), reactive nitroxide species, immunoactive enzymes (e.g. ARG, indoleamine 2,3‐dioxygenase, aminopeptidase), and prostaglandin E2 (PGE2).^104^ In preclinical murine models of colitis and primary sclerosing cholangitis (PSC), the gut microbiome was shown to induce hepatocyte secretion of CXCL1, which recruits chemokine (C‐X‐C motif) receptor (CXCR)2^+^ PMN‐MDSCs and forms an immunosuppressive environment.^105^ PSC is an autoimmune disease of the bile ducts and an important risk factor for CCA; approximately 50% of CCAs are diagnosed within the first year of a PSC diagnosis.^106^ A separate study found that anti‐PD‐L1 therapy alone failed to control orthotopic CCA in mice^58^; however, depleting both PMN‐MDSCs and TAMs enhanced the therapeutic response, suggesting that targeting PMN‐MDSCs may improve ICI efficacy in CCA.^58^

TAMs contribute to chronic inflammatory carcinogenesis by releasing proinflammatory mediators such as IL‐1β, IL‐6 and reactive nitrogen/oxygen intermediates.^107^ They suppress T‐cell responses by overexpressing PD‐L1, ARG1, PGE2 and TGF‐β, and promote CCL22‐mediated Treg accumulation.^108^ Arlauckas *et al*.^109^ demonstrated that Fcγ receptor‐expressing bone marrow‐derived TAMs can capture anti‐PD‐1 antibodies from the surface of T cells, leading to PD‐1 inhibitor resistance. In addition, in non‐small‐cell lung cancer (NSCLC), T cell dysfunction associated with co‐expression of inhibitory receptors including PD‐1, CTLA‐4 and lymphocyte‐activation gene 3 drives disease progression and ICI resistance.^110^ These findings led to Food and Drug Administration (FDA) approval of ICI combinations, including pembrolizumab, nivolumab and ipilimumab, for advanced NSCLC,^111^, ^112^ highlighting a potentially translatable strategy for CCA.

Collectively, immunosuppressive cells and molecules present within the TME represent a major barrier to ICI efficacy in CCA. Targeting these components may be critical to improving patient responses and overcoming therapeutic resistance.

## Manipulating the cholangiocarcinoma immune phenotype to improve response to immune checkpoint inhibitors

### Targeting immunosuppressive cell populations

Efforts to convert a ‘cold’ TME into an inflamed or ‘hot’ TME have been explored in various cancers, including CCA, to improve immunotherapy responsiveness. Given their predominance and importance within the CCA TME, targeting immunosuppressive TAMs and MDSCs represents a promising therapeutic approach.^10^ In CCA xenograft mouse models, CSF1R inhibition blocked monocyte‐to‐macrophage differentiation, decreased tumor cell proliferation and increased apoptosis, ultimately lowering tumor burden.^55^ Similarly, blockade of monocyte chemoattractant protein 1 (MCP‐1), an activator of TAMs, reduced TAM recruitment and tumor growth in CCA xenograft models.^113^ GM‐CSF, an important cytokine in myeloid cell programming, has been associated with decreased OS in patients with resected iCCA.^114^ Inhibition of GM‐CSF reduced TAM abundance, repolarised TAMs and MDSCs to facilitate increased T cell responses and slowed tumor growth in an orthotopic mouse CCA model.^114^

Tavazoie and colleagues^115^ revealed that agonism of the liver‐X nuclear receptor (LXR) reduced MDSC levels in both mouse models and patients during a first‐in‐human dose escalation phase I trial. LXR activation caused transcriptional upregulation of apolipoprotein E, impairing MDSC survival and enhancing CD8^+^ T cell activation across several cancers, including lung, melanoma and colon.^115^ These results support LXR agonists as potential agents to enhance ICI therapy in ‘cold’ tumors such as CCA.

In a murine CCA model, Loeuillard *et al*.^58^ found that MDSC inhibition using anti‐Ly6G antibody or the LXR agonist GW3965, combined with TAM depletion via anti‐CSF1R antibody, improved anti‐PD‐1 efficacy, extending survival and reducing tumor progression. Importantly, TAM blockade alone led to a compensatory emergence of G‐MDSCs, negating its antitumor effect, highlighting the importance of dual inhibition of both MDSCs and TAMs.^58^ Another study identified a cellular crosstalk mechanism that limited anti‐CSF1R efficacy,^116^ providing an explanation for the previous findings.^58^ Specifically, tumor‐derived CSF1 induced histone deacetylase 2‐mediated suppression of granulocytic chemokine expression by CAFs, restricting granulocyte migration.^116^ CSF1R inhibition reversed this effect, resulting in an accumulation of PMN‐MDSCs.^116^

Dual targeting of immunosuppressive TAMs and MDSCs may overcome compensatory immunosuppressive mechanisms and enhance ICI response in CCA. This strategy warrants further investigations in clinical trials.

### Modulating cytokine signalling

Another plausible target is the cytokine TGF‐β, which has been found to promote CCA initiation and progression.^117^ Huang *et al*.^117^ revealed that inhibiting TGF‐β1 suppressed CCA growth, whereas its overexpression resulted in the opposite effect and even induced intrahepatic metastasis in a rat model. An *in vitro* study further suggested that targeting cytokine receptors of IL‐10 and TGF‐β on DCs enhanced the cytolytic activity of DC‐activated CD8^+^ T cells against CCA cell lines,^118^ supporting this strategy as a means to boost ICI efficacy in ‘cold’ tumors. Lustri and colleagues^119^ evaluated two TGF‐β1 inhibitors in primary human mucin‐ and mixed‐intrahepatic CCA cell cultures, finding that one reduced cell viability and induced apoptosis, while the other impaired cell migration. Targeting TGF‐β signalling through two different axes, via a combination of both inhibitors, was proposed to more effectively disrupt tumor survival and induce apoptosis of human CCA cells.^119^

However, clinical translation of TGF‐β‐targeted therapies has so far been limited. A phase I open‐label trial of bintrafusp alfa, a bifunctional fusion protein that targets both TGF‐β and PD‐L1, in 30 patients with advanced HCC demonstrated a manageable safety profile and preliminary efficacy.^120^ An expanded phase II trial with 159 CCA patients was discontinued after failing to meet its primary endpoint of improving OS, despite reporting an ORR of 10.1% in the 9‐month follow‐up.^121^ Nonetheless, novel TGF‐β1 inhibitors in combination with ICIs remain a promising area for future clinical trials to potentially enhance CCA response to immunotherapy.

Cytokine infusion therapy is another up‐and‐coming immunotherapeutic approach under investigation for CCA, which involves administering endogenous or engineered cytokines to directly modulate the TME.^122^ A phase II trial demonstrated a potential synergistic effect of combining cytokine infusion with ICIs.^123^ Compared with pembrolizumab monotherapy, the addition of intravenous GM‐CSF improved efficacy, because of increased antigen presentation and DC recruitment.^123^ Despite enhanced efficacy, the trial did not meet its ORR endpoint, and adverse events occurred in 69% of patients.^124^

### Immune activation approaches

Activation and priming of CD8^+^ T cells using CD40 agonists to stimulate macrophages and DCs is another rational approach to improve ICI responses. In murine iCCA models, combination therapy with anti‐CD40 and anti‐PD‐1 antibodies significantly decreased tumor burden compared to either monotherapy.^125^ This was accompanied by increased infiltration of macrophages, DCs, and effector immune cells including CD8^+^/ CD4^+^ T cells and NK cells.^125^ CD40 agonism in combination with ICIs may provide an effective therapeutic avenue for CCA. Interestingly, dual CD40/PD‐1 blockade significantly improved response to gemcitabine‐cisplatin, extending OS compared to chemotherapy alone.^125^

Activation of the stimulator of interferon genes (STING) pathway is another way to convert ‘cold’ tumors into ‘hot’ ones by promoting lymphocyte infiltration and TAM repolarisation.^126^, ^127^ One study investigated STING expression and its role in iCCA.^128^ Immunohistochemical analysis of FFPE tissue from 44 patients revealed that high STING expression in early‐stage iCCA correlated with longer OS.^128^ STING agonism in murine iCCA models exerted stage‐specific antitumor effects, with diminished efficacy in advanced‐stage tumors associated with increased PD‐L1 expression.^128^ However, combining a STING agonist with anti‐PD‐L1 therapy significantly reduced tumor burden in these advanced tumors.^128^ The authors proposed STING monotherapy to induce immune cell infiltration in early‐stage iCCA, and STING plus ICIs for advanced‐stage iCCA.^128^

Despite these promising findings, clinical translation may be limited by the fact that most CCA cases are diagnosed at advanced stages.^11^ Nonetheless, these preclinical studies lay theoretical foundations for future trials exploring STING agonists and CD40‐based therapies in CCA.

### Adoptive cell therapy

Adoptive cell therapy is an emerging therapeutic option for CCA that involves the transfer of host‐ or donor‐derived immune cells to promote antitumor responses. For instance, TIL therapy involves isolating and expanding patient lymphocytes *in vitro* before reinfusing them into the patient to enhance immune‐mediated tumor clearance.^129^ Another method involves genetically engineering immune cells to express chimeric antigen receptors (CARs) specific to tumor antigens. While most CAR‐based research has focused on T cells, recent studies have explored CAR‐engineered NK cells because of their lack of prior sensitisation and favorable safety profile.^130^

Given the association between immune ‘hot’ tumors and improved prognosis, efforts are underway to evaluate whether direct infusion of lymphocytes via TIL therapy can enhance antitumor immunity in CCA. Although both CD8^+^ T cells and NK cells exhibit *in vitro* cytotoxicity against CCA cells, evidence for their efficacy *in vivo* remains limited, despite FDA approval of adoptive cell therapies for other malignancies such as melanoma.^131^

One case study reported that a patient with metastatic CCA achieved 13 months of disease stabilisation following infusion of mutation‐specific CD4^+^ T cells.^85^ A recent phase I/II clinical trial evaluating pembrolizumab in combination with allogeneic NK cells in CCA patients found an improved ORR of 17.4% compared with pembrolizumab monotherapy, with no increase in adverse events.^43^ These studies reflect the potential of TIL therapy, particularly when mutation‐targeted or in combination with ICIs, as a therapeutic approach for CCA. However, additional research is required to evaluate long‐term safety and efficacy relative to existing first‐line therapies.

## Preclinical models for cholangiocarcinoma research

### Current limitations in preclinical models

A major challenge in understanding CCA tumor biology, immunology and developing clinically translatable treatments is the lack of preclinical models that accurately replicate the CCA TME and disease.^132^ Over 90% of drugs fail phase I clinical trials, with the main reasons being lack of clinical efficacy (40–50%), unmanageable toxicity (30%) and poor drug‐like properties (10–15%).^133^ Of these, poor clinical efficacy is the most common reason for failure, driving a renewed focus on improving preclinical models for evaluating novel compounds,^133^ a priority highlighted in the 2020 CCA consensus statement.^134^ Developing translational models for CCA is difficult because of the inter‐ and intra‐tumoral heterogeneity across disease subtypes.^135^ While animal models provide a complex, dynamic *in vivo* system to study CCA, biological differences often limit the translatability of findings, contributing to the 97% failure rate of oncology drugs.^133^, ^135^, ^136^, ^137^ Research into the CCA TME and immune phenotype modulation requires models with an intact TME, yet many animal models use immunodeficient hosts (e.g. xenograft, or patient‐derived xenografts), limiting their utility.^138^ Ethical concerns around animal welfare have driven the push for researchers to implement the 3Rs: reduction, refinement and replacement of animal models.^139^, ^140^ These translational and ethical limitations of animal research have increased efforts to develop and use patient‐derived preclinical models for CCA research and treatment development.

### Advanced immunological preclinical models

**Figure 3:**
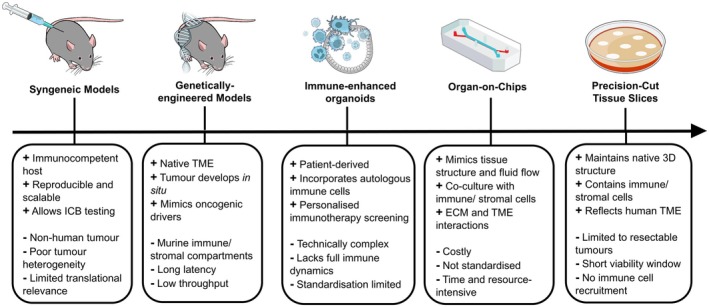
Immunologically relevant preclinical models for studying the CCA TME and immunotherapy. This figure summarises the strengths and limitations of five advanced preclinical models used to investigate immune modulation and tumor‐immune interactions in CCA. Syngeneic mouse models allow immunotherapy testing in an immunocompetent host but lack human tumor components. Genetically engineered models (GEMs) replicate tumor initiation *in situ* with a native TME, though are limited by murine‐specific biology and long development times. Immune‐enhanced organoids incorporate autologous immune cells into patient‐derived cultures, enabling personalised immunotherapy studies, but are technically complex and lack full immune dynamics. Organ‐on‐chip platforms support co‐culture of tumor, stromal and immune cells under physiologically relevant flow, yet face standardisation and scalability challenges. Human precision‐cut tissue slices (hPCTS) preserve 3D architecture and resident immune cells, providing a highly translational platform, although viability is limited, and immune cell recruitment cannot be modelled without additional supplementation.

There are various animal and 3D culture models of CCA, including syngeneic models, organoids and human precision‐cut tissue slices (hPCTS) with differing strengths and weaknesses,^135^ shown in Figure 3. Research into the TME requires models that contain immune cells and ECM, while accurately recapitulating CCA 3D architecture.^135^ Cellular interactions between tumor cells, the ECM and the immune system are crucial for disease progression, tumor angiogenesis and treatment response.^97^, ^141^, ^142^

Animal models offer valuable insight into tumor biology and cellular interactions within a complex, physiological organism. Unlike xenograft models using immunocompromised hosts, syngeneic models use mice with a functioning immune system. One study established several murine CCA cell lines implanted into murine livers, forming tumors with phenotypic characteristics of human CCA, including desmoplasia and cytokeratin‐19 expression.^143^ This approach models tumor‐stroma‐immune interactions and may provide an alternative platform to conduct immunotherapeutic studies,^135^ though species differences in tumor biology and immunology pose a significant limitation.

Genetically engineered models (GEMs) can overcome species differences by enabling animal models to exhibit human CCA‐associated genetic mutations and biochemical, proteomic and phenotypic characteristics.^144^ Generating GEMs involves activating oncogenes and/or inactivating tumor‐suppressor genes *in vivo* using transgenic and gene‐targeting approaches.^145^ Transgenic models introduce foreign DNA via retroviral delivery or embryo microinjection,^146^, ^147^ enabling the study of mutations such as KRAS activation,^148^ PTEN deletion^149^ and TP53 loss.^150^ One study using a TP53 deletion successfully modelled chronic liver injury with inflammation and fibrosis before iCCA developed,^151^ while other studies found PTEN and KRAS models did not develop chronic liver injury or inflammation prior to iCCA formation.^152^, ^153^ Hydrodynamic transfection delivers DNA to liver cells via rapid tail vein injection, offering a flexible, cost‐effective method that better reflects sporadic gene expression seen in human CCA.^154^ GEMs may advance CCA immunology research by revealing how specific gene alterations shape the TME and tumor‐ECM interactions, critical for understanding immune evasion and developing targeted immunotherapies. However, GEMs may exhibit unpredictable transgene expression and require complex, time‐consuming breeding.^145^, ^155^

Organoid models provide a more physiologically representative tissue model than 2D cultures or spheroids by incorporating organ‐specific cell types that display certain organ functions.^156^ These self‐organising and ‐renewing 3D models are formed by embedding patient‐derived epithelial stem cells in a 3D cellular matrix.^156^ Traditional 3D organoid models typically replicate only the tumor epithelium and lack immune cells, limiting their ability to simulate the TME and immunotherapy response.^157^ Tumor‐immune organoid models have been developed in colorectal cancer,^158^ NSCLC,^158^ pancreatic cancer^159^ and CCA,^160^ containing patient‐matched immune cells. Zhou *et al*.^160^ demonstrated that CCA organoids co‐cultured with peripheral blood mononuclear cells (PBMCs) or T cells induced effective contact‐based and soluble factor‐mediated cytotoxicity. Such co‐culture methods may support the development of personalised ICI treatments and evaluation of novel therapies. However, the absence of an ECM, including stromal tissue and vasculature, limits their ability to replicate full cellular dynamics that influence therapeutic response and cancer progression.^161^

Organ‐on‐chips are microfluidic chips that grow engineered or natural miniature tissues.^162^ One study developed an *in vitro* 3D microfluidic iCCA model by co‐culturing primary iCCA cells, CAFs and endothelial cells for up to 4 days.^163^ This platform reliably mimicked the *in vivo* iCCA microenvironment and established cross‐talk between cell types, leading to ECM remodelling, and may serve as a tool for personalised medicine.^163^ However, standardisation of design is limited, and the manufacturing and operation of this model are costly in time, resources and money, with its physiological relevance compared to other models yet to be validated.^162^

### Human precision‐cut tissue slices for translational immunotherapy research

An *ex vivo* model gaining interest is hPCTS. These patient‐derived tissue slices retain parenchymal and non‐parenchymal cells at physiological ratios and orientation, preserve *in vivo* 3D tissue structure, and can be derived from different organs,^135^ including CCA.^164^ hPCTS, particularly in the context of liver disease, has been tested for toxicity assessment, disease modelling and drug development.^135^ Brugger *et al*.^165^ revealed that hPCTS could be used to study hepatocyte cell death *ex vivo*, using immunohistochemical staining of apoptotic markers including cleaved‐caspase‐3. One study using hPCTS to model liver fibrosis and test anti‐fibrotic drugs identified major limitations: reduced viability beyond 48 h and non‐physiological oxygen conditions.^166^ However, mathematical modelling shows that physiologically relevant oxygen gradients can be achieved in 5‐mm‐diameter slices at atmospheric oxygen concentrations using an air–liquid interface.^167^

CCA hPCTS remain viable for up to 15 days in culture, confirmed by MTS cell viability assays and the absence of cleaved‐caspase‐3 staining.^164^, ^168^ HCC hPCTS have been shown to preserve tumor morphology, stroma and tumor‐infiltrated immune cells.^169^ Combination treatments of doxorubicin, regorafenib with anti‐PD‐1 therapy and monotherapies reduced cell proliferation,^169^ while the immune phenotype was maintained for up to 8 days in culture, supporting their potential for preclinical chemo‐ and immunotherapy testing.^169^

Further investigation of immune cell activation status within hPCTS is needed to better define the model's strengths and limitations; such studies are ongoing in our laboratory. hPCTS are generated from resectable CCA specimens collected from patients eligible for surgery with curative intent, limiting their use in modelling earlier or unresectable, advanced disease. While this restricts their utility for evaluating therapies in advanced CCA, they provide a physiologically relevant snapshot of immune cell populations within the human CCA TME. However, hPCTS cannot replicate physiological immune cell trafficking from circulation into tissue,^170^, ^171^ limiting their ability to evaluate adaptive immune responses to ICI therapy. This has been partially addressed in pancreatic ductal adenocarcinoma hPCTS, where the migration of adoptively transferred leukocytes was observed, following the addition of autologous PBMCs, splenocytes or genetically engineered macrophages.^172^, ^173^ This demonstrates another promising application of hPCTS in testing novel adoptive cellular immunotherapies,^173^ including CAR T‐cell therapy.

## Conclusions and future directions

Immunotherapy resistance remains a major challenge in CCA treatment, warranting further investigations into the role of immunosuppressive cells in contributing to a ‘cold’ TME and its poor responsiveness to current ICIs. To our knowledge, efforts to modulate the CCA TME and shift the immune phenotype have largely relied on animal models or simplified *in vitro* cultures, both of which have significant ethical and translational limitations. The limited success of clinical trials so far likely reflects the constrained translational potential of these preclinical platforms.

Despite their own limitations, hPCTS could be a valuable platform for modelling human CCA and its TME. These tissue slices retain the complex architecture and cellular composition of the native tumor, making them ideal for studying immune interactions within the TME and testing pharmacological strategies aimed at converting ‘cold’ tumors into ‘hot’ tumors. This model could ultimately support the development of more effective immunotherapeutic strategies and facilitate a personalised medicine framework for CCA, enabling the creation of tailored treatment modalities for individual patients. Moreover, hPCTS may serve as a valuable tool in drug discovery and development, offering mechanistic insights into therapeutic responses in a clinically relevant human model of CCA.

## Author contributions

**Maria‐Danae Jessel:** Conceptualization; visualization; writing – original draft; writing – review and editing. **Owen McGreevy:** Visualization; writing – original draft; writing – review and editing. **Lauryn McReynolds:** Writing – original draft. **Lekh N Dahal:** Supervision; writing – review and editing. **Timothy Gilbert:** Writing – review and editing. **Hassan Z Malik:** Supervision; writing – review and editing. **Christopher E Goldring:** Supervision; writing – review and editing. **Laura E Randle:** Funding acquisition; supervision; writing – review and editing.

## Conflict of interest

LER reports article publishing charges were provided by the University of Liverpool. LER reports a relationship with the National Centre for the Replacement, Refinement and Reduction of Animals in Research that includes funding grants and membership on the NC3Rs PhD studentship assessment board. CEG reports financial support by the University of Liverpool. MDJ, OM, LR, LND, TG and HZM declare that they have no known competing financial interests or personal relationships that could have appeared to influence the work reported in this paper.

## References

[1] Banales JM , Marin JJG , Lamarca A *et al*. Cholangiocarcinoma 2020: the next horizon in mechanisms and management. Nat Rev Gastroenterol Hepatol 2020; 17: 557–588. 32606456 10.1038/s41575-020-0310-z PMC7447603

[2] Valle JW , Kelley KR , Nervi B , Oh D‐Y , Zhu AX . Biliary tract cancer. Lancet 2021; 397: 428–444. 33516341 10.1016/S0140-6736(21)00153-7

[3] Khan AS , Dageforde LA . Cholangiocarcinoma. Surg Clin North Am 2019; 99: 315–335. 30846037 10.1016/j.suc.2018.12.004

[4] Banales JM , Cardinale V , Carpino G *et al*. Cholangiocarcinoma: current knowledge and future perspectives consensus statement from the European network for the study of Cholangiocarcinoma (ENS‐CCA). Nat Rev Gastroenterol Hepatol 2016; 13: 261–280. 27095655 10.1038/nrgastro.2016.51

[5] Collaboration GBoDC . The global burden of cancer 2013. JAMA Oncol 2015; 1: 505–527. 26181261 10.1001/jamaoncol.2015.0735 PMC4500822

[6] Cai S , Sivakumar S . The 11th revision of the international statistical classification of disease and related health problems and Cholangiocarcinoma. Hepatobiliary Surg Nutr 2022; 11: 276–279. 35464287 10.21037/hbsn-22-69 PMC9023819

[7] Qurashi M , Vithayathil M , Khan SA . Epidemiology of cholangiocarcinoma. Eur J Surg Oncol 2025; 51: 107064. 37709624 10.1016/j.ejso.2023.107064

[8] Organization WH . International Classification of Diseases 11th Revision. 2025. https://icd.who.int/en/ (accessed 4 July 2025).

[9] NCRAS . Get data out – liver and biliary tract. 2022. https://nhsd‐ndrs.shinyapps.io/get_data_out/ (accessed 15th May 2025).

[10] Ilyas SI , Affo S , Goyal L *et al*. Cholangiocarcinoma — novel biological insights and therapeutic strategies. Nat Rev Clin Oncol 2023; 20: 470–486. 37188899 10.1038/s41571-023-00770-1 PMC10601496

[11] Lee YT , Wang JJ , Luu M *et al*. Comparison of clinical features and outcomes between intrahepatic Cholangiocarcinoma and hepatocellular carcinoma in the United States. Hepatology 2021; 74: 2622–2632. 34114675 10.1002/hep.32007

[12] Chan K‐M , Tsai C‐Y , Yeh C‐N *et al*. Characterization of intrahepatic cholangiocarcinoma after curative resection: outcome, prognostic factor, and recurrence. BMC Gastroenterol 2018; 18: 180. 30514231 10.1186/s12876-018-0912-x PMC6278092

[13] Ebata T , Hirano S , Konishi M *et al*. Randomized clinical trial of adjuvant gemcitabine chemotherapy versus observation in resected bile duct cancer. Br J Surg 2018; 105: 192–202. 29405274 10.1002/bjs.10776

[14] Edeline J , Benabdelghani M , Bertaut A *et al*. Gemcitabine and oxaliplatin chemotherapy or surveillance in resected biliary tract cancer (PRODIGE 12‐ACCORD 18‐UNICANCER GI): a randomized phase III study. J Clin Oncol 2019; 37: 658–667. 30707660 10.1200/JCO.18.00050

[15] Primrose JN , Fox RP , Palmer DH *et al*. Capecitabine compared with observation in resected biliary tract cancer (BILCAP): a randomised, controlled, multicentre, phase 3 study. Lancet Oncol 2019; 20: 663–673. 30922733 10.1016/S1470-2045(18)30915-X

[16] Shroff RT , Kennedy EB , Bachini M *et al*. Adjuvant therapy for resected biliary tract cancer: ASCO clinical practice guideline. J Clin Oncol 2019; 37: 1015–1027. 30856044 10.1200/JCO.18.02178

[17] Valle J , Wasan H , Palmer DH *et al*. Cisplatin plus gemcitabine versus gemcitabine for biliary tract cancer. N Engl J Med 2010; 362: 1273–1281. 20375404 10.1056/NEJMoa0908721

[18] NICE . Durvalumab with gemcitabine and cisplatin for treating unresectable or advanced biliary tract cancer. 2024. https://www.nice.org.uk/guidance/ta944 (accessed 4th October 2024). 40198028

[19] Oh D‐Y , He AR , Qin S *et al*. Durvalumab plus gemcitabine and cisplatin in advanced biliary tract cancer. N Engl J Med Evid 2022; 1: EVIDoa2200015. 10.1056/EVIDoa2200015 38319896

[20] Nakamura H , Arai Y , Totoki Y *et al*. Genomic spectra of biliary tract cancer. Nat Genet 2015; 47: 1003–1010. 26258846 10.1038/ng.3375

[21] Sabbatino F , Villani V , Yearley JH *et al*. PD‐L1 and HLA class I antigen expression and clinical course of the disease in intrahepatic Cholangiocarcinoma. Clin Cancer Res 2016; 22: 470–478. 26373575 10.1158/1078-0432.CCR-15-0715 PMC5296951

[22] Zhou G , Sprengers D , Mancham S *et al*. Reduction of immunosuppressive tumor microenvironment in cholangiocarcinoma by *ex vivo* targeting immune checkpoint molecules. J Hepatol 2019; 71: 753–762. 31195061 10.1016/j.jhep.2019.05.026

[23] Fournel L , Wu Z , Stadler N *et al*. Cisplatin increases PD‐L1 expression and optimizes immune check‐point blockade in non‐small cell lung cancer. Cancer Lett 2019; 464: 5–14. 31404614 10.1016/j.canlet.2019.08.005

[24] Pei Q , Pan J , Zhu H *et al*. Gemcitabine‐treated pancreatic cancer cell medium induces the specific CTL antitumor activity by stimulating the maturation of dendritic cells. Int Immunopharmacol 2014; 19: 10–16. 24389382 10.1016/j.intimp.2013.12.022

[25] Oh D‐Y , Lee K‐H , Lee D‐W *et al*. Gemcitabine and cisplatin plus durvalumab with or without tremelimumab in chemotherapy‐naive patients with advanced biliary tract cancer: an open‐label, single‐centre, phase 2 study. Lancet Gastroenterol Hepatol 2022; 7: 522–532. 35278356 10.1016/S2468-1253(22)00043-7

[26] Greten TF , Schwabe R , Bardeesy N *et al*. Immunology and immunotherapy of cholangiocarcinoma. Nat Rev Gastroenterol Hepatol 2023; 20: 349–365. 36697706 10.1038/s41575-022-00741-4 PMC12468729

[27] Kelley RK , Ueno M , Yoo C *et al*. Pembrolizumab in combination with gemcitabine and cisplatin compared with gemcitabine and cisplatin alone for patients with advanced biliary tract cancer (KEYNOTE‐966): a randomised, double‐blind, placebo‐controlled, phase 3 trial. Lancet 2023; 401: 1853–1865. 37075781 10.1016/S0140-6736(23)00727-4

[28] NICE . Pembrolizumab with gemcitabine and cisplatin for untreated advanced biliary tract cancer (terminated appraisal). 2024. https://www.nice.org.uk/guidance/ta966 (accessed 4th October 2024). 40127318

[29] Yarchoan M , Cope L , Ruggieri AN *et al*. Multicenter randomized phase II trial of atezolizumab with or without cobimetinib in biliary tract cancers. J Clin Invest 2021; 131: e152670. 34907910 10.1172/JCI152670 PMC8670844

[30] Doki Y , Ueno M , Hsu CH *et al*. Tolerability and efficacy of durvalumab, either as monotherapy or in combination with tremelimumab, in patients from Asia with advanced biliary tract, esophageal, or head‐and‐neck cancer. Cancer Med 2022; 11: 2550–2560. 35611499 10.1002/cam4.4593 PMC9249982

[31] Kim RD , Chung V , Alese OB *et al*. A phase 2 multi‐institutional study of Nivolumab for patients with advanced refractory biliary tract cancer. JAMA Oncol 2020; 6: 888–894. 32352498 10.1001/jamaoncol.2020.0930 PMC7193528

[32] Piha‐Paul SA , Oh DY , Ueno M *et al*. Efficacy and safety of pembrolizumab for the treatment of advanced biliary cancer: Results from the KEYNOTE‐158 and KEYNOTE‐028 studies. Int J Cancer 2020; 147: 2190–2198. 32359091 10.1002/ijc.33013

[33] Goetze T , Gonzalez‐Carmona MA , Kochen L *et al*. ADJUBIL: phase II study of adjuvant immunotherapy with STRIDE regimen with/without capecitabine in biliary tract cancers. Future Oncol 2024; 20: 307–315. 38410920 10.2217/fon-2023-0961

[34] Klein O , Kee D , Nagrial A *et al*. Evaluation of combination Nivolumab and Ipilimumab immunotherapy in patients with advanced biliary tract cancers: Subgroup analysis of a phase 2 nonrandomized clinical trial. JAMA Oncol 2020; 6: 1405–1409. 32729929 10.1001/jamaoncol.2020.2814 PMC7393585

[35] Sahai V , Griffith KA , Beg MS *et al*. A randomized phase 2 trial of nivolumab, gemcitabine, and cisplatin or nivolumab and ipilimumab in previously untreated advanced biliary cancer: BilT‐01. Cancer 2022; 128: 3523–3530. 35895381 10.1002/cncr.34394 PMC9540241

[36] Chapin WJ , Agarwal P , DiCicco L *et al*. Phase II trial of XmAb20717 (vudalimab) in patients with advanced biliary tract cancers. J Clin Oncol 2023; 41: TPS4184‐TPS.

[37] Chen X , Wu X , Wu H *et al*. Camrelizumab plus gemcitabine and oxaliplatin (GEMOX) in patients with advanced biliary tract cancer: a single‐arm, open‐label, phase II trial. J Immunother Cancer 2020; 8: 8. 10.1136/jitc-2020-001240 PMC7656907 33172881

[38] Ueno M , Ikeda M , Morizane C *et al*. Nivolumab alone or in combination with cisplatin plus gemcitabine in Japanese patients with unresectable or recurrent biliary tract cancer: a non‐randomised, multicentre, open‐label, phase 1 study. Lancet Gastroenterol Hepatol 2019; 4: 611–621. 31109808 10.1016/S2468-1253(19)30086-X

[39] Feng K , Liu Y , Zhao Y *et al*. Efficacy and biomarker analysis of nivolumab plus gemcitabine and cisplatin in patients with unresectable or metastatic biliary tract cancers: results from a phase II study. J Immunother Cancer 2020; 8: e000367. 32487569 10.1136/jitc-2019-000367 PMC7269541

[40] Fan S , Gai C , Li B , Wang G . Efficacy and safety of envafolimab in the treatment of advanced dMMR/MSI‐H solid tumors: a single‐arm meta‐analysis. Oncol Lett 2023; 26: 351. 37545619 10.3892/ol.2023.13937 PMC10398626

[41] Robert C . A decade of immune‐checkpoint inhibitors in cancer therapy. Nat Commun 2020; 11: 3801. 32732879 10.1038/s41467-020-17670-y PMC7393098

[42] Xiong J , Wang Q‐Q . Mechanisms and strategies to overcome immunotherapy resistance in hepatobiliary malignancies. Hepatobiliary Pancreat Dis Int 2022; 21: 430–439. 35907687 10.1016/j.hbpd.2022.07.006

[43] Leem G , Jang S‐I , Cho J‐H *et al*. Safety and efficacy of allogeneic natural killer cells in combination with Pembrolizumab in patients with chemotherapy‐refractory biliary tract cancer: a multicenter open‐label phase 1/2a trial. Cancers (Basel) 2022; 14: 4229. 36077766 10.3390/cancers14174229 PMC9454779

[44] Affo S , Yu L‐X , Schwabe RF . The role of cancer‐associated fibroblasts and fibrosis in liver cancer. Annu Rev Pathol 2017; 12: 153–186. 27959632 10.1146/annurev-pathol-052016-100322 PMC5720358

[45] Desbois M , Wang Y . Cancer‐associated fibroblasts: key players in shaping the tumor immune microenvironment. Immunol Rev 2021; 302: 241–258. 34075584 10.1111/imr.12982

[46] Ellis MJ , Singer C , Hornby A , Rasmussen A , Cullen KJ . Insulin‐like growth factor mediated stromal‐epithelial interactions in human breast cancer. Breast Cancer Res Treat 1994; 31: 249–261. 7881103 10.1007/BF00666158

[47] San Francisco IF , DeWolf WC , Peehl DM , Olumi AF . Expression of transforming growth factor‐beta 1 and growth in soft agar differentiate prostate carcinoma‐associated fibroblasts from normal prostate fibroblasts. Int J Cancer 2004; 112: 213–218. 15352032 10.1002/ijc.20388

[48] Chuaysri C , Thuwajit P , Paupairoj A , Chau‐In S , Suthiphongchai T , Thuwajit C . Alpha‐smooth muscle actin‐positive fibroblasts promote biliary cell proliferation and correlate with poor survival in cholangiocarcinoma. Oncol Rep 2009; 21: 957–969. 19287994 10.3892/or_00000309

[49] Kitano Y , Okabe H , Yamashita Y‐i *et al*. Tumour‐infiltrating inflammatory and immune cells in patients with extrahepatic cholangiocarcinoma. Br J Cancer 2018; 118: 171–180. 29123259 10.1038/bjc.2017.401 PMC5785749

[50] Ma C , Zhang Q , Greten TF . MDSCs in liver cancer: a critical tumor‐promoting player and a potential therapeutic target. Cell Immunol 2021; 361: 104295. 33508529 10.1016/j.cellimm.2021.104295 PMC7882059

[51] Zimmer CL , Filipovic I , Cornillet M *et al*. Mucosal‐associated invariant T‐cell tumor infiltration predicts long‐term survival in cholangiocarcinoma. Hepatology 2022; 75: 1154–1168. 34719787 10.1002/hep.32222

[52] Hasita H , Komohara Y , Okabe H *et al*. Significance of alternatively activated macrophages in patients with intrahepatic cholangiocarcinoma. Cancer Sci 2010; 101: 1913–1919. 20545696 10.1111/j.1349-7006.2010.01614.x PMC11158749

[53] Paillet J , Kroemer G , Pol JG . Immune contexture of cholangiocarcinoma. Curr Opin Gastroenterol 2020; 36: 70–76. 31895228 10.1097/MOG.0000000000000613

[54] Sun D , Luo T , Dong P *et al*. M2‐polarized tumor‐associated macrophages promote epithelial‐mesenchymal transition via activation of the AKT3/PRAS40 signaling pathway in intrahepatic cholangiocarcinoma. J Cell Biochem 2020; 121: 2828–2838. 31692069 10.1002/jcb.29514

[55] Boulter L , Guest RV , Kendall TJ *et al*. WNT signaling drives cholangiocarcinoma growth and can be pharmacologically inhibited. J Clin Invest 2015; 125: 1269–1285. 25689248 10.1172/JCI76452 PMC4362247

[56] Loilome W , Bungkanjana P , Techasen A *et al*. Activated macrophages promote Wnt/β‐catenin signaling in cholangiocarcinoma cells. Tumour Biol 2014; 35: 5357–5367. 24549785 10.1007/s13277-014-1698-2 PMC4862210

[57] Thanee MLW , Techasen A , Namwat N , Boonmars T , Pairojkul C , Yongvanit P . Quantitative changes in tumor‐associated M2 macrophages characterize Cholangiocarcinoma and their association with metastasis. Asian Pac J Cancer Prev 2015; 16: 3043–3050. 25854403 10.7314/apjcp.2015.16.7.3043

[58] Loeuillard E , Yang J , Buckarma E *et al*. Targeting tumor‐associated macrophages and granulocytic myeloid‐derived suppressor cells augments PD‐1 blockade in cholangiocarcinoma. J Clin Invest 2020; 130: 5380–5396. 32663198 10.1172/JCI137110 PMC7524481

[59] Highfill SL , Cui Y , Giles AJ *et al*. Disruption of CXCR2‐mediated MDSC tumor trafficking enhances anti‐PD1 efficacy. Sci Transl Med 2014; 6: 237ra67. 10.1126/scitranslmed.3007974 PMC6980372 24848257

[60] Fridlender ZG , Sun J , Kim S *et al*. Polarization of tumor‐associated neutrophil phenotype by TGF‐β: “N1” versus “N2” TAN. Cancer Cell 2009; 16: 183–194. 19732719 10.1016/j.ccr.2009.06.017 PMC2754404

[61] Zhou S‐L , Dai Z , Zhou Z‐J *et al*. CXCL5 contributes to tumor metastasis and recurrence of intrahepatic cholangiocarcinoma by recruiting infiltrative intratumoral neutrophils. Carcinogenesis 2013; 35: 597–605. 24293410 10.1093/carcin/bgt397

[62] Gu F‐M , Gao Q , Shi G‐M *et al*. Intratumoral IL‐17+ cells and neutrophils show strong prognostic significance in intrahepatic Cholangiocarcinoma. Ann Surg Oncol 2012; 19: 2506–2514. 22411204 10.1245/s10434-012-2268-8

[63] Mao ZY , Zhu GQ , Xiong M , Ren L , Bai L . Prognostic value of neutrophil distribution in cholangiocarcinoma. World J Gastroenterol 2015; 21: 4961–4968. 25945010 10.3748/wjg.v21.i16.4961 PMC4408469

[64] Zhou Z , Wang P , Sun R *et al*. Tumor‐associated neutrophils and macrophages interaction contributes to intrahepatic cholangiocarcinoma progression by activating STAT3. J Immunother Cancer 2021; 9: e001946. 33692217 10.1136/jitc-2020-001946 PMC7949476

[65] Konishi D , Umeda Y , Yoshida K *et al*. Regulatory T cells induce a suppressive immune milieu and promote lymph node metastasis in intrahepatic cholangiocarcinoma. Br J Cancer 2022; 127: 757–765. 35597869 10.1038/s41416-022-01838-y PMC9381563

[66] Qian Y , Yao W , Yang T *et al*. aPKC‐ι/P‐Sp1/snail signaling induces epithelial–mesenchymal transition and immunosuppression in cholangiocarcinoma. Hepatology 2017; 66: 1165–1182. 28574228 10.1002/hep.29296

[67] Löffler MW , Chandran PA , Laske K *et al*. Personalized peptide vaccine‐induced immune response associated with long‐term survival of a metastatic cholangiocarcinoma patient. J Hepatol 2016; 65: 849–855. 27397612 10.1016/j.jhep.2016.06.027 PMC5756536

[68] Ma C , Peng C , Lu X *et al*. Downregulation of FOXP3 inhibits invasion and immune escape in cholangiocarcinoma. Biochem Biophys Res Commun 2015; 458: 234–239. 25623530 10.1016/j.bbrc.2015.01.067

[69] Cornillet M , Jansson H , Schaffer M *et al*. Imbalance of genes encoding natural killer immunoglobulin‐like receptors and human leukocyte antigen in patients with biliary cancer. Gastroenterology 2019; 157: 1067–1080. 31229495 10.1053/j.gastro.2019.06.023

[70] Oliviero B , Varchetta S , Mele D *et al*. MICA/B‐targeted antibody promotes NK cell–driven tumor immunity in patients with intrahepatic cholangiocarcinoma. Onco Targets Ther 2022; 11: 2035919. 10.1080/2162402X.2022.2035919 PMC8865231 35223192

[71] Fukuda Y , Asaoka T , Eguchi H *et al*. Endogenous CXCL9 affects prognosis by regulating tumor‐infiltrating natural killer cells in intrahepatic cholangiocarcinoma. Cancer Sci 2020; 111: 323–333. 31799781 10.1111/cas.14267 PMC7004525

[72] Gentilini A , Pastore M , Marra F , Raggi C . The role of stroma in Cholangiocarcinoma: The intriguing interplay between fibroblastic component, immune cell subsets and tumor epithelium. Int J Mol Sci 2018; 19: 2885. 30249019 10.3390/ijms19102885 PMC6213545

[73] Morisaki T , Umebayashi M , Kiyota A *et al*. Combining cetuximab with killer lymphocytes synergistically inhibits human cholangiocarcinoma cells*in vitro* . Anticancer Res 2012; 32: 2249–2256. 22641659

[74] Banchereau J , Steinman RM . Dendritic cells and the control of immunity. Nature 1998; 392: 245–252. 9521319 10.1038/32588

[75] Takagi S , Miyagawa S‐I , Ichikawa E *et al*. Dendritic cells, T‐cell infiltration, and grp94 expression in cholangiocellular carcinoma. Hum Pathol 2004; 35: 881–886. 15257553 10.1016/j.humpath.2004.03.016

[76] Wang J , Ilyas S . Targeting the tumor microenvironment in cholangiocarcinoma: implications for therapy. Expert Opin Investig Drugs 2021; 30: 429–438. 10.1080/13543784.2021.1865308 PMC8096665 33322977

[77] Thepmalee C , Aussara P , Jatuporn S *et al*. Suppression of TGF‐β and IL‐10 receptors on self‐differentiated dendritic cells by short‐hairpin RNAs enhanced activation of effector T‐cells against cholangiocarcinoma cells. Hum Vaccin Immunother 2020; 16: 2318–2327. 31976810 10.1080/21645515.2019.1701913 PMC7644170

[78] Panya A , Thepmalee C , Sawasdee N *et al*. Cytotoxic activity of effector T cells against cholangiocarcinoma is enhanced by self‐differentiated monocyte‐derived dendritic cells. Cancer Immunol Immunother 2018; 67: 1579–1588. 30056600 10.1007/s00262-018-2212-2 PMC11028072

[79] Sadeghlar F , Vogt A , Mohr RU *et al*. Induction of cytotoxic effector cells towards cholangiocellular, pancreatic, and colorectal tumor cells by activation of the immune checkpoint CD40/CD40L on dendritic cells. Cancer Immunol Immunother 2021; 70: 1451–1464. 33180184 10.1007/s00262-020-02746-x PMC8053193

[80] Kasper HU , Drebber U , Stippel DL , Dienes HP , Gillessen A . Liver tumor infiltrating lymphocytes: comparison of hepatocellular and cholangiolar carcinoma. World J Gastroenterol 2009; 15: 5053–5057. 19859998 10.3748/wjg.15.5053 PMC2768884

[81] Chang SH . T helper 17 (Th17) cells and interleukin‐17 (IL‐17) in cancer. Arch Pharm Res 2019; 42: 549–559. 30941641 10.1007/s12272-019-01146-9

[82] Chraa D , Naim A , Olive D , Badou A . T lymphocyte subsets in cancer immunity: friends or foes. J Leukoc Biol 2018; 105: 243–255. 30387907 10.1002/JLB.MR0318-097R

[83] Su S‐B , Zhang J‐F , Huang F‐F , Cen Y , Jiang H‐X . Large numbers of interleukins‐22‐ and ‐17A‐producing T helper cells in cholangiocarcinoma related to liver fluke infection. Microbiol Immunol 2017; 61: 345–354. 28718957 10.1111/1348-0421.12500

[84] Walker JA , McKenzie ANJ . TH2 cell development and function. Nat Rev Immunol 2018; 18: 121–133. 29082915 10.1038/nri.2017.118

[85] Tran E , Turcotte S , Gros A *et al*. Cancer immunotherapy based on mutation‐specific CD4+ T cells in a patient with epithelial cancer. Science 2014; 344: 641–645. 24812403 10.1126/science.1251102 PMC6686185

[86] Liu D , Heij LR , Czigany Z *et al*. The role of tumor‐infiltrating lymphocytes in cholangiocarcinoma. J Exp Clin Cancer Res 2022; 41: 127. 35392957 10.1186/s13046-022-02340-2 PMC8988317

[87] Mao Z‐Y , Zhu G‐Q , Xiong M , Ren L , Bai L . Prognostic value of neutrophil distribution in cholangiocarcinoma. World J Gastroenterol 2015; 21: 4961–4968. 25945010 10.3748/wjg.v21.i16.4961 PMC4408469

[88] Xia T , Li K , Niu N *et al*. Immune cell atlas of cholangiocarcinomas reveals distinct tumor microenvironments and associated prognoses. J Hematol Oncol 2022; 15: 37. 35346322 10.1186/s13045-022-01253-z PMC8962046

[89] Zhu Y , Wang XY , Zhang Y *et al*. Programmed death ligand 1 expression in human intrahepatic cholangiocarcinoma and its association with prognosis and CD8+ T‐cell immune responses. Cancer Manag Res 2018; 10: 4113–4123. 30323667 10.2147/CMAR.S172719 PMC6174308

[90] Tian L , Ma J , Ma L *et al*. PD‐1/PD‐L1 expression profiles within intrahepatic cholangiocarcinoma predict clinical outcome. World J Surg Oncol 2020; 18: 303. 33228682 10.1186/s12957-020-02082-5 PMC7686719

[91] Cao H , Huang T , Dai M *et al*. Tumor microenvironment and its implications for antitumor immunity in Cholangiocarcinoma: future perspectives for novel therapies. Int J Biol Sci 2022; 18: 5369–5390. 36147461 10.7150/ijbs.73949 PMC9461676

[92] Hegde PS , Karanikas V , Evers S . The where, the when, and the how of immune monitoring for cancer immunotherapies in the era of checkpoint inhibition. Clin Cancer Res 2016; 22: 1865–1874. 27084740 10.1158/1078-0432.CCR-15-1507

[93] Chen DS , Mellman I . Elements of cancer immunity and the cancer–immune set point. Nature 2017; 541: 321–330. 28102259 10.1038/nature21349

[94] Galon J , Bruni D . Approaches to treat immune hot, altered and cold tumours with combination immunotherapies. Nat Rev Drug Discov 2019; 18: 197–218. 30610226 10.1038/s41573-018-0007-y

[95] Hegde PS , Chen DS . Top 10 challenges in cancer immunotherapy. Immunity 2020; 52: 17–35. 31940268 10.1016/j.immuni.2019.12.011

[96] Job S , Rapoud D , Dos Santos A *et al*. Identification of four immune subtypes characterized by distinct composition and functions of tumor microenvironment in intrahepatic Cholangiocarcinoma. Hepatology 2020; 72: 965–981. 31875970 10.1002/hep.31092 PMC7589418

[97] Fabris L , Perugorria MJ , Mertens J *et al*. The tumour microenvironment and immune milieu of cholangiocarcinoma. Liver Int 2019; 39: 63–78. 30907492 10.1111/liv.14098 PMC10878127

[98] Bai R , Chen N , Li L *et al*. Mechanisms of cancer resistance to immunotherapy. Front Oncol 2020; 10: 1290. 32850400 10.3389/fonc.2020.01290 PMC7425302

[99] Gao J , Shi LZ , Zhao H *et al*. Loss of IFN‐γ pathway genes in tumor cells as a mechanism of resistance to anti‐CTLA‐4 therapy. Cell 2016; 167: 397–404. 27667683 10.1016/j.cell.2016.08.069 PMC5088716

[100] Chen G , Huang AC , Zhang W *et al*. Exosomal PD‐L1 contributes to immunosuppression and is associated with anti‐PD‐1 response. Nature 2018; 560: 382–386. 30089911 10.1038/s41586-018-0392-8 PMC6095740

[101] Marvel D , Gabrilovich DI . Myeloid‐derived suppressor cells in the tumor microenvironment: expect the unexpected. J Clin Invest 2015; 125: 3356–3364. 26168215 10.1172/JCI80005 PMC4588239

[102] Ugel S , De Sanctis F , Mandruzzato S , Bronte V . Tumor‐induced myeloid deviation: when myeloid‐derived suppressor cells meet tumor‐associated macrophages. J Clin Invest 2015; 125: 3365–3376. 26325033 10.1172/JCI80006 PMC4588310

[103] Condamine T , Ramachandran I , Youn J‐I , Gabrilovich DI . Regulation of tumor metastasis by myeloid‐derived suppressor cells. Annu Rev Med 2015; 66: 97–110. 25341012 10.1146/annurev-med-051013-052304 PMC4324727

[104] Condamine T , Gabrilovich DI . Molecular mechanisms regulating myeloid‐derived suppressor cell differentiation and function. Trends Immunol 2011; 32: 19–25. 21067974 10.1016/j.it.2010.10.002 PMC3053028

[105] Zhang Q , Ma C , Duan Y *et al*. Gut microbiome directs hepatocytes to recruit MDSCs and promote Cholangiocarcinoma. Cancer Discov 2021; 11: 1248–1267. 33323397 10.1158/2159-8290.CD-20-0304 PMC8102309

[106] Boberg KM , Bergquist A , Mitchell S *et al*. Cholangiocarcinoma in primary Sclerosing cholangitis: risk factors and clinical presentation. Scand J Gastroenterol 2002; 37: 1205–1211. 12408527 10.1080/003655202760373434

[107] Mantovani A , Allavena P , Sica A , Balkwill F . Cancer‐related inflammation. Nature 2008; 454: 436–444. 18650914 10.1038/nature07205

[108] Noy R , Pollard JW . Tumor‐associated macrophages: from mechanisms to therapy. Immunity 2014; 41: 49–61. 25035953 10.1016/j.immuni.2014.06.010 PMC4137410

[109] Arlauckas SP , Garris CS , Kohler RH *et al*. *In vivo* imaging reveals a tumor‐associated macrophage–mediated resistance pathway in anti–PD‐1 therapy. Sci Transl Med 2017; 9: eaal3604. 28490665 10.1126/scitranslmed.aal3604 PMC5734617

[110] Thommen DS , Schreiner J , Müller P *et al*. Progression of lung cancer is associated with increased dysfunction of T cells defined by coexpression of multiple inhibitory receptors. Cancer Immunol Res 2015; 3: 1344–1355. 26253731 10.1158/2326-6066.CIR-15-0097

[111] Gandhi L , Rodríguez‐Abreu D , Gadgeel S *et al*. Pembrolizumab plus chemotherapy in metastatic non–small‐cell lung cancer. N Engl J Med 2018; 378: 2078–2092. 29658856 10.1056/NEJMoa1801005

[112] Hellmann MD , Paz‐Ares L , Caro RB *et al*. Nivolumab plus Ipilimumab in advanced non–small‐cell lung cancer. N Engl J Med 2019; 381: 2020–2031. 31562796 10.1056/NEJMoa1910231

[113] Dwyer BJ , Jarman EJ , Gogoi‐Tiwari J *et al*. TWEAK/Fn14 signalling promotes cholangiocarcinoma niche formation and progression. J Hepatol 2021; 74: 860–872. 33221352 10.1016/j.jhep.2020.11.018

[114] Ruffolo LI , Jackson KM , Kuhlers PC *et al*. GM‐CSF drives myelopoiesis, recruitment and polarisation of tumour‐associated macrophages in cholangiocarcinoma and systemic blockade facilitates antitumour immunity. Gut 2022; 71: 1386–1398. 34413131 10.1136/gutjnl-2021-324109 PMC8857285

[115] Tavazoie MF , Pollack I , Tanqueco R *et al*. LXR/ApoE activation restricts innate immune suppression in cancer. Cell 2018; 172: 825–840. 29336888 10.1016/j.cell.2017.12.026 PMC5846344

[116] Kumar V , Donthireddy L , Marvel D *et al*. Cancer‐associated fibroblasts neutralize the anti‐tumor effect of CSF1 receptor blockade by inducing PMN‐MDSC infiltration of tumors. Cancer Cell 2017; 32: 654–668. 29136508 10.1016/j.ccell.2017.10.005 PMC5827952

[117] Huang C‐K , Aihara A , Iwagami Y *et al*. Expression of transforming growth factor β1 promotes cholangiocarcinoma development and progression. Cancer Lett 2016; 380: 153–162. 27364974 10.1016/j.canlet.2016.05.038 PMC4973469

[118] Thepmalee C , Panya A , Junking M , Chieochansin T , Yenchitsomanus P‐t . Inhibition of IL‐10 and TGF‐β receptors on dendritic cells enhances activation of effector T‐cells to kill cholangiocarcinoma cells. Hum Vaccin Immunother 2018; 14: 1423–1431. 29420117 10.1080/21645515.2018.1431598 PMC6037468

[119] Lustri AM , Di Matteo S , Fraveto A *et al*. TGF‐β signaling is an effective target to impair survival and induce apoptosis of human cholangiocarcinoma cells: a study on human primary cell cultures. PLoS One 2017; 12: e0183932. 28873435 10.1371/journal.pone.0183932 PMC5584931

[120] Doi T , Fujiwara Y , Koyama T *et al*. Phase I study of the Bifunctional fusion protein Bintrafusp Alfa in Asian patients with advanced solid tumors, including a hepatocellular carcinoma safety‐assessment cohort. Oncologist 2020; 25: e1292–e1302. 32324927 10.1634/theoncologist.2020-0249 PMC7485354

[121] Oh D‐Y , de Braud F , Bridgewater J *et al*. P5‐5 phase 2/3 study of bintrafusp alfa with gemcitabine plus cisplatin as first‐line treatment of biliary tract cancer. Ann Oncol 2021; 32: S333.

[122] Song K . Current development status of cytokines for cancer immunotherapy. Biomol Ther (Seoul) 2024; 32: 13–24. 38148550 10.4062/biomolther.2023.196 PMC10762268

[123] Kelley RK , Mitchell E , Behr S *et al*. Phase II trial of pembrolizumab (PEM) plus granulocyte macrophage colony stimulating factor (GM‐CSF) in advanced biliary cancers (ABC). J Clin Oncol 2018; 36: 386.

[124] Kelley RK , Bracci PM , Keenan B *et al*. Pembrolizumab (PEM) plus granulocyte macrophage colony stimulating factor (GM‐CSF) in advanced biliary cancers (ABC): Final outcomes of a phase 2 trial. J Clin Oncol 2022; 40: 444.

[125] Diggs LP , Ruf B , Ma C *et al*. CD40‐mediated immune cell activation enhances response to anti‐PD‐1 in murine intrahepatic cholangiocarcinoma. J Hepatol 2021; 74: 1145–1154. 33276030 10.1016/j.jhep.2020.11.037 PMC9662232

[126] Jiang M , Chen P , Wang L *et al*. cGAS‐STING, an important pathway in cancer immunotherapy. J Hematol Oncol 2020; 13: 81. 32571374 10.1186/s13045-020-00916-z PMC7310007

[127] Dahal LN , Dou L , Hussain K *et al*. STING activation reverses lymphoma‐mediated resistance to antibody immunotherapy. Cancer Res 2017; 77: 3619–3631. 28512240 10.1158/0008-5472.CAN-16-2784 PMC5500176

[128] Luo S , Li S , Liu C *et al*. Stage‐specificity of STING activation in intrahepatic cholangiocarcinoma determines the efficacy of its agonism. Cancer Lett 2024; 594: 216992. 38797231 10.1016/j.canlet.2024.216992

[129] Zhao Y , Deng J , Rao S *et al*. Tumor infiltrating lymphocyte (TIL) therapy for solid tumor treatment: progressions and challenges. Cancers (Basel) 2022; 14: 4160. 36077696 10.3390/cancers14174160 PMC9455018

[130] Peng L , Sferruzza G , Yang L , Zhou L , Chen S . CAR‐T and CAR‐NK as cellular cancer immunotherapy for solid tumors. Cell Mol Immunol 2024; 21: 1089–1108. 39134804 10.1038/s41423-024-01207-0 PMC11442786

[131] Klobuch S , Seijkens TTP , Schumacher TN , Haanen JBAG . Tumour‐infiltrating lymphocyte therapy for patients with advanced‐stage melanoma. Nat Rev Clin Oncol 2024; 21: 173–184. 38191921 10.1038/s41571-023-00848-w

[132] Kamb A . What's wrong with our cancer models? Nat Rev Drug Discov 2005; 4: 161–165. 15688078 10.1038/nrd1635

[133] Sun D , Gao W , Hu H , Zhou S . Why 90% of clinical drug development fails and how to improve it? Acta Pharm Sin B 2022; 12: 3049–3062. 35865092 10.1016/j.apsb.2022.02.002 PMC9293739

[134] Calvisi DF , Boulter L , Vaquero J *et al*. Criteria for preclinical models of cholangiocarcinoma: scientific and medical relevance. Nat Rev Gastroenterol Hepatol 2023; 20: 462–480. 36755084 10.1038/s41575-022-00739-y

[135] McGreevy O , Bosakhar M , Gilbert T *et al*. The importance of preclinical models in cholangiocarcinoma. Eur J Surg Oncol 2024; 51: 108304. 38653585 10.1016/j.ejso.2024.108304

[136] Lin A , Giuliano CJ , Palladino A *et al*. Off‐target toxicity is a common mechanism of action of cancer drugs undergoing clinical trials. Sci Transl Med 2019; 11: eaaw8412. 31511426 10.1126/scitranslmed.aaw8412 PMC7717492

[137] Pound P . Are animal models needed to discover, develop and test pharmaceutical drugs for humans in the 21st century? Animals 2020; 10: 2455. 33371480 10.3390/ani10122455 PMC7767523

[138] Mohr R , Özdirik B , Knorr J *et al*. *In vivo* models for cholangiocarcinoma—what can we learn for human disease? Int J Mol Sci 2020; 21: 4993. 32679791 10.3390/ijms21144993 PMC7404171

[139] The principles of humane experimental technique. Med J Aust 1960; 1: 500.

[140] Mukherjee P , Roy S , Ghosh D , Nandi SK . Role of animal models in biomedical research: a review. Lab Anim Res 2022; 38: 18. 35778730 10.1186/s42826-022-00128-1 PMC9247923

[141] Chen Z , Guo P , Xie X , Yu H , Wang Y , Chen G . The role of tumour microenvironment: a new vision for cholangiocarcinoma. J Cell Mol Med 2019; 23: 59–69. 30394682 10.1111/jcmm.13953 PMC6307844

[142] Wang M , Zhao J , Zhang L *et al*. Role of tumor microenvironment in tumorigenesis. J Cancer 2017; 8: 761–773. 28382138 10.7150/jca.17648 PMC5381164

[143] Ilyas SI , Fischbach SR , Bronk SF *et al*. YAP‐associated chromosomal instability and cholangiocarcinoma in mice. Oncotarget 2018; 9: 5892–5905. 29464042 10.18632/oncotarget.23638 PMC5814182

[144] Loeuillard E , Fischbach SR , Gores GJ , Ilyas SI . Animal models of cholangiocarcinoma. Biochim Biophys Acta (BBA) – Mol Basis Dis 2019; 1865: 982–992. 10.1016/j.bbadis.2018.03.026 PMC6177316 29627364

[145] He L , Tian DA , Li PY , He XX . Mouse models of liver cancer: progress and recommendations. Oncotarget 2015; 6: 23306–23322. 26259234 10.18632/oncotarget.4202 PMC4695120

[146] Massa A , Varamo C , Vita F *et al*. Evolution of the experimental models of cholangiocarcinoma. Cancers (Basel) 2020; 12: 2308. 32824407 10.3390/cancers12082308 PMC7463907

[147] Kumar TR , Larson M , Wang H , McDermott J , Bronshteyn I . Transgenic mouse technology: principles and methods. In: Park‐Sarge O‐K , Curry TE , eds. Molecular Endocrinology: Methods and Protocols. Totowa, NJ: Humana Press; 2009:335–362. 10.1007/978-1-60327-378-7_22 PMC4095860 19763515

[148] Huang L , Guo Z , Wang F , Fu L . KRAS mutation: from undruggable to druggable in cancer. Signal Transduct Target Ther 2021; 6: 386. 34776511 10.1038/s41392-021-00780-4 PMC8591115

[149] Lee Y‐R , Chen M , Pandolfi PP . The functions and regulation of the PTEN tumour suppressor: new modes and prospects. Nat Rev Mol Cell Biol 2018; 19: 547–562. 29858604 10.1038/s41580-018-0015-0

[150] Hassin O , Oren M . Drugging p53 in cancer: one protein, many targets. Nat Rev Drug Discov 2023; 22: 127–144. 36216888 10.1038/s41573-022-00571-8 PMC9549847

[151] Farazi PA , Zeisberg M , Glickman J , Zhang Y , Kalluri R , DePinho RA . Chronic bile duct injury associated with fibrotic matrix microenvironment provokes cholangiocarcinoma in p53‐deficient mice. Cancer Res 2006; 66: 6622–6627. 16818635 10.1158/0008-5472.CAN-05-4609

[152] Xu X , Kobayashi S , Qiao W *et al*. Induction of intrahepatic cholangiocellular carcinoma by liver‐specific disruption of Smad4 and Pten in mice. J Clin Invest 2006; 116: 1843–1852. 16767220 10.1172/JCI27282 PMC1474816

[153] Ikenoue T , Terakado Y , Nakagawa H *et al*. Correction: Corrigendum: a novel mouse model of intrahepatic cholangiocarcinoma induced by liver‐specific Kras activation and Pten deletion. Sci Rep 2017; 7: 39567. 28045050 10.1038/srep39567 PMC5206645

[154] Chen X , Calvisi DF . Hydrodynamic transfection for generation of novel mouse models for liver cancer research. Am J Pathol 2014; 184: 912–923. 24480331 10.1016/j.ajpath.2013.12.002 PMC3969989

[155] Erice O , Vallejo A , Ponz‐Sarvise M *et al*. Genetic mouse models as *In vivo* tools for cholangiocarcinoma research. Cancers (Basel) 2019; 11: 1868. 31769429 10.3390/cancers11121868 PMC6966555

[156] Lancaster MA , Knoblich JA . Organogenesis in a dish: Modeling development and disease using organoid technologies. Science 2014; 345: 1247125. 25035496 10.1126/science.1247125

[157] Polak R , Zhang ET , Kuo CJ . Cancer organoids 2.0: modelling the complexity of the tumour immune microenvironment. Nat Rev Cancer 2024; 24: 523–539. 38977835 10.1038/s41568-024-00706-6

[158] Dijkstra KK , Cattaneo CM , Weeber F *et al*. Generation of tumor‐reactive T cells by Co‐culture of peripheral blood lymphocytes and tumor organoids. Cell 2018; 174: 1586–1598. 30100188 10.1016/j.cell.2018.07.009 PMC6558289

[159] Meng Q , Xie S , Gray GK *et al*. Empirical identification and validation of tumor‐targeting T cell receptors from circulation using autologous pancreatic tumor organoids. J Immunother Cancer 2021; 9: 9. 10.1136/jitc-2021-003213 PMC8601084 34789550

[160] Zhou G , Lieshout R , van Tienderen GS *et al*. Modelling immune cytotoxicity for cholangiocarcinoma with tumour‐derived organoids and effector T cells. Br J Cancer 2022; 127: 649–660. 35597867 10.1038/s41416-022-01839-x PMC9381772

[161] van Tienderen GS , Groot Koerkamp B , IJzermans JNM , van der Laan LJW , Verstegen MMA . Recreating tumour complexity in a dish: organoid models to study liver cancer cells and their extracellular environment. Cancers (Basel) 2019; 11: 1706. 31683901 10.3390/cancers11111706 PMC6896153

[162] Leung CM , de Haan P , Ronaldson‐Bouchard K *et al*. A guide to the organ‐on‐a‐chip. Nat Rev Methods Primers 2022; 2: 33.

[163] Polidoro MA , Saladino G , Ferrari E , Rasponi M , Marzorati S , Lleo A . Cholangiocarcinoma‐on‐chip: a 3D liver tumor platform for personalized medicine. Dig Liver Dis 2023; 55: S8.

[164] Gilbert T , Randle L , Diaz‐neito R *et al*. Developing a patient‐derived model of cholangiocarcinoma using precision cut tissue slices (PCTS). Eur J Surg Oncol 2024; 50: 107758.

[165] Brugger M , Laschinger M , Lampl S *et al*. High precision‐cut liver slice model to study cell‐autonomous antiviral defense of hepatocytes within their microenvironment. JHEP Rep 2022; 4: 100465. 35462860 10.1016/j.jhepr.2022.100465 PMC9019249

[166] Paish HL , Reed LH , Brown H *et al*. A bioreactor technology for modeling fibrosis in human and rodent precision‐cut liver slices. Hepatology 2019; 70: 1377–1391. 30963615 10.1002/hep.30651 PMC6852483

[167] Chidlow SJ , Randle LE , Kelly RA . Predicting physiologically‐relevant oxygen concentrations in precision‐cut liver slices using mathematical modelling. PLoS One 2022; 17: e0275788. 36322567 10.1371/journal.pone.0275788 PMC9629643

[168] McGreevy O , Gilbert T , Jessel M *et al*. A preclinical model of human liver using precision cut tissue slice culture [version 1; peer review: awaiting peer review]. F1000Res 2025; 14: 571. 40777732 10.12688/f1000research.162495.1 PMC12329406

[169] Jagatia R , Doornebal EJ , Rastovic U *et al*. Patient‐derived precision cut tissue slices from primary liver cancer as a potential platform for preclinical drug testing. EBioMedicine 2023; 97: 104826. 37806285 10.1016/j.ebiom.2023.104826 PMC10667128

[170] Liu Y , Wu P , Wang Y *et al*. Application of precision‐cut lung slices as an *in vitro* model for research of inflammatory respiratory diseases. Bioengineering (Basel) 2022; 9: 767. 36550973 10.3390/bioengineering9120767 PMC9774555

[171] Nizamoglu M , Joglekar MM , Almeida CR *et al*. Innovative three‐dimensional models for understanding mechanisms underlying lung diseases: powerful tools for translational research. Eur Respir Rev 2023; 32: 230042. 37495250 10.1183/16000617.0042-2023 PMC10369168

[172] Brempelis KJ , Cowan CM , Kreuser SA *et al*. Genetically engineered macrophages persist in solid tumors and locally deliver therapeutic proteins to activate immune responses. J Immunother Cancer 2020; 8: e001356. 33115946 10.1136/jitc-2020-001356 PMC7594542

[173] Jiang X , Seo YD , Chang JH *et al*. Long‐lived pancreatic ductal adenocarcinoma slice cultures enable precise study of the immune microenvironment. Onco Targets Ther 2017; 6: e1333210. 10.1080/2162402X.2017.1333210 PMC5543820 28811976

